# Reliability and agreement of CBCT-based alveolar bone assessments for follow-up studies on adolescent orthodontic patients using multiplanar reconstruction and various CBCT units

**DOI:** 10.1186/s40510-026-00637-y

**Published:** 2026-07-21

**Authors:** Linda Bokander Matilainen, Kristina Johansson, Liselotte Paulsson, Helena Christell

**Affiliations:** 1 Department of Orthodontics, Faculty of Odontology, Malmö University, Malmo, Sweden; 2 Department of Orthodontics, Östersunds Hospital, Östersund, Sweden; 3 Department of Radiology, Helsingborg Hospital, Helsingborg, Sweden

**Keywords:** Cone-beam computed tomography, Reliability, Agreement, Alveolar bone, Fenestration, Orthodontic treatment

## Abstract

**Background:**

CBCT is increasingly used to assess alveolar bone in orthodontic patients; however, reliability and agreement for detecting changes in follow-up assessments remain uncertain. This study evaluated the reliability and agreement of linear marginal alveolar bone level measurements, and agreement in detecting fenestrations in CBCT volumes acquired from different units before and after active orthodontic treatment in adolescents, using a multiplanar reconstruction-based measurement protocol for follow-up studies.

**Methods:**

CBCT volumes (n = 96) from 48 adolescents (mean age 14.4 years) enrolled in a randomised controlled trial on fixed-appliance treatment for crowding were acquired at baseline and posttreatment using five distinct CBCT units after objective image-quality testing. Buccal and lingual bone levels at the central incisors, canines, and second premolars were measured from the cementoenamel junction to the alveolar crest according to a predefined protocol. Three independent raters performed linear measurements, two assessed fenestrations greater than 2.2 mm, and one repeated all measurements. Reliability was evaluated using intraclass correlation coefficients. Agreement for the bone level measurements was assessed using Dahlberg’s formula and visualised with Bland–Altman plots. Minimal detectable change was calculated per site, and percentage agreement was used for fenestration detection.

**Results:**

Intrarater reliability ranged from moderate to excellent at both time points. Dahlberg’s random errors ranged from 0.19 to 0.71 mm at baseline, and 0.19 to 1.07 mm posttreatment. Interrater reliability ranged from poor to good, with random errors ranging from 0.23 to 2.54 mm. Bland–Altman plots revealed minor systematic bias for the intrarater measurements. Minimal detectable changes demonstrated the ability to detect clinically relevant alveolar bone changes. Agreement for fenestration detection was high (intrarater ≥ 81%, interrater ≥ 74%).

**Conclusion:**

The protocol demonstrated intrarater reliability and agreement suitable for assessing clinically relevant changes in marginal bone levels and for detecting fenestrations in follow-up studies of adolescents treated orthodontically for crowding without extraction. However, the results were influenced by rater variability and imaging conditions and were most reliable when the assessments were performed by the same rater. Assessment of reliability and agreement under study-specific conditions is recommended. The findings highlight both the potential and methodological challenges of CBCT-based alveolar bone assessments, offering implications for clinical practice and future research in this field.

*Trial registration*: CROWDIT: ClinicalTrials.gov (NCT05664282).

**Supplementary Information:**

The online version contains supplementary material available at 10.1186/s40510-026-00637-y.

## Background

Assessment of alveolar bone height, including preoperative implant planning, evaluation of periodontal treatment outcomes, and orthodontic treatment planning, is important in both clinical practice and research [1].

Reductions in alveolar bone height and thickness are recognised adverse effects of orthodontic treatment and appear to be more pronounced at the buccal and lingual surfaces than at the proximal surfaces [2]. Cone-beam computed tomography (CBCT) provides high-resolution images in three planes for assessments of the alveolar bone. While studies have reported reliable and accurate linear measurements of marginal bone levels in CBCT [3, 4], others have presented contradictory results [5].

The measurability and precision of CBCT-based alveolar bone assessments are influenced by image quality, which is affected by spatial resolution, voxel size, and field of view (FOV) [6], as well as patient positioning within the FOV [7–9], viewing mode [10], variability between CBCT devices [11], motion during CBCT acquisition [9, 12], and bone thickness [13]. CBCT may overestimate bone loss, both in linear measurements and in the detection of fenestrations, defined as an area of root surface denuded of bone without involvement of the alveolar bone margin [14]. Hence, both technical and biological factors may influence measurement results, particularly when assessing thin alveolar bone using CBCT [15]. The inclusion of multiple CBCT units from different manufacturers in research studies may therefore require unit-specific adjustment of exposure settings to achieve comparable image quality while also adhering to the ALADA (As Low As Diagnostically Acceptable) principle [16].

Reliability can be described as the ratio of between-subject variability to total variability [17]. Reported outcomes vary with study design, including the use of Plexiglas plates [18], dry skulls with or without soft tissue substitutes [3] and markers such as drilled holes [19], 3D rendering [20], and the methodology of preselected images [5]. Although good accuracy and reliability of linear bone level measurements have been demonstrated [3, 21], in vitro and ex vivo studies do not reflect in vivo conditions, such as patients moving during acquisition [9], or the influence of bone remodelling during orthodontic treatment [5, 22]. Thus, reliability is not inherent to an instrument or method but depends on the context in which it is tested, including the raters, environment, testing conditions and population characteristics [23].

To enable repeatable measurements across CBCT examinations in follow-up studies using different CBCT systems, a standardised measurement protocol based on the identification of anatomical landmarks is needed. Multiplanar reconstruction (MPR)-based measurement protocols for follow-up studies have demonstrated excellent interrater reliability in a sample of periodontally affected central incisors. The cases were restricted to CBCT volumes free of artefacts [24] such as beam hardening. In clinical follow-up settings, such artefacts, together with orthodontic treatment-related bone changes [25], as well as tooth eruption and growth, may complicate the measurement procedure.

The aim of this study was to evaluate the reliability and agreement of linear marginal bone level measurements at incisors, canines, and premolars, and to assess agreement in detecting fenestrations, using an MPR-based measurement protocol for follow‑up studies applied to CBCT volumes acquired in vivo from adolescent orthodontic patients, using different CBCT units.

We hypothesised that reliability and agreement would be suitable for the intended application of linear alveolar bone measurements and fenestration assessment, although lower than those reported in previous studies conducted under controlled conditions, e.g. using phantoms, markers, or preselected images.

## Methods

### Trial design, patients and ethics

This methodological reliability and agreement study using in vivo data followed the Guidelines for Reporting Reliability and Agreement Studies (GRRAS) [26]. It was conducted within the Crowded Displaced Teeth (CROWDIT) project, which evaluates the outcomes of non-extraction fixed appliance treatment in adolescents with crowded and displaced teeth and is registered at ClinicalTrials.gov (NCT05664282).

CROWDIT is a multicentre, two-arm, parallel-group randomised controlled trial (RCT) with a 1:1 allocation ratio. Eligible participants were adolescents (12–17 years) with dental crowding and irregularity. After providing assent and informed consent, participants were randomised to treatment with either a passive self-ligating bracket system (Damon Q^™^, 0.022 variable torque, Ormco Corporation, Orange, CA, USA) or a conventional bracket system (Victory low profile APC plus^™^, 0.022 MBT standard torque, 3M, St Paul, MN, USA) at one of three orthodontic clinics in Sweden: a university clinic, a private practice, and a clinic in regional care. Eligibility criteria, demographics, treatment protocols, and clinical outcomes have been reported previously [27].

For the present reliability and agreement study, CROWDIT participants with available CBCT examinations at both baseline (T0) and post-active treatment (T1) with acceptable visibility of the alveolar bone were eligible for inclusion. A sample corresponding to the calculated sample size (see below) was randomly selected from the eligible cohort using a computer-generated sequence.

The study was approved by the Regional Ethical Review Board in Lund (Dnr. 2014/647) and the Swedish Radiation Safety Authority, through the radiation protection committees of Skåne University Hospital and Dalarna County Council, in accordance with the Declaration of Helsinki. No additional radiological examinations were performed for this study.

### CBCT acquisition

Prior to trial initiation, calibration and dose optimisation were performed at each radiology clinic using the SEDENTEXCT Quality Control Phantom (Leeds Test Objects Ltd, North Yorkshire, UK) by an oral and maxillofacial radiologist according to the described methodology [21]. The exposure parameters were established based on objective quality in relation to radiation dose in accordance with the ALADA principle [16]. The protocols varied with respect to tube voltage (80–120 kV), tube current (2–6 mA), exposure time (4–17.5 s), rotation (180–360°), voxel size (125-300 µm), and FOV (8 × 8–8 × 16 cm) (Table 1). Radiological examinations were acquired to include both jaws, using five CBCT units representing four models across three radiology clinics (A, B, and C), following the predetermined acquisition protocols.

**Table 1:** CBCT equipment, acquisition parameters and distribution of CBCT examinations

Clinic		CBCT machine	n	Voltage (kV)	Tube current (mA)	Exposure time (s)	Rotation (°)	FOV (cm)	Voxel (µm)	Software
T0	A	3D Accuitomo^®^170	9	80	3/6	17.5	360	8 × 8	160	i-Dixel^®^ (Morita, Kyoto, JP)
	A	Veraviewepocs^®^	14	80/90	3/5	9.3/9.4	180	8 × 8	125	i-Dixel^®^ (Morita, Kyoto, JP)
	B	i-CAT^®^ 9140	20	120	5	4	360	8 × 16	300	Romexis^®^ (Planmeca, Helsinki, FI)
	C	3D Accuitomo^®^170	5	80	3	17.5	360	8 × 8	160	i-Dixel^®^ (Morita, Kyoto, JP)
T1	A	Veraviewepocs^®^	23	80/90	2/3/5	9.3/9.4	180	8 × 8	125	i-Dixel^®^ (Morita, Kyoto, JP)
	B	i-CAT^®^ 9140	7	120	5	4	360	8 × 16	300	Romexis^®^ (Planmeca, Helsinki, FI)
	B	ProMax^®^ 3D Mid	13	90	5/6	12	200	8 × 8	200	Romexis^®^ (Planmeca, Helsinki, FI)
	C	3D Accuitomo^®^170	5	80	3	17.5	360	8 × 8	160	i-Dixel^®^ (Morita, Kyoto, JP)

*Clinic* Radiology clinic; *CBCT* cone-beam computed tomography; *n* number of CBCT examinations acquired; *kV* kilovolts; *mA* milliamperes; *s* seconds; *FOV* field of view; *cm* centimetres; *μm* micrometres; *T0* baseline; *T1* post-active treatment; *JP* Japan; *FI* Finland

### Data processing, workstation and equipment

All CBCT volumes were imported as Digital Imaging and Communications in Medicine files, ensuring full dataset integrity on the workstation in clinic A. Image analysis was performed under dimmed ambient lighting conditions. Examinations from clinics A and C were evaluated using i-Dixel^®^ software (J. Morita, Kyoto Japan), whereas those from clinic B were assessed using Romexis^®^ (Planmeca Oy, Helsinki, Finland) (Table 1). A 27-inch monochrome LCD flat-panel monitor (3840 × 2160; Olorin MCD271, Olorin AB, Kungsbacka, Sweden) was used for all assessments. Contrast and brightness were standardised using a reference image prepared by an oral and maxillofacial radiologist (HC). No time restrictions were imposed during the evaluation process.

### Raters, reformatting and measurement protocol

**Fig. 1:**
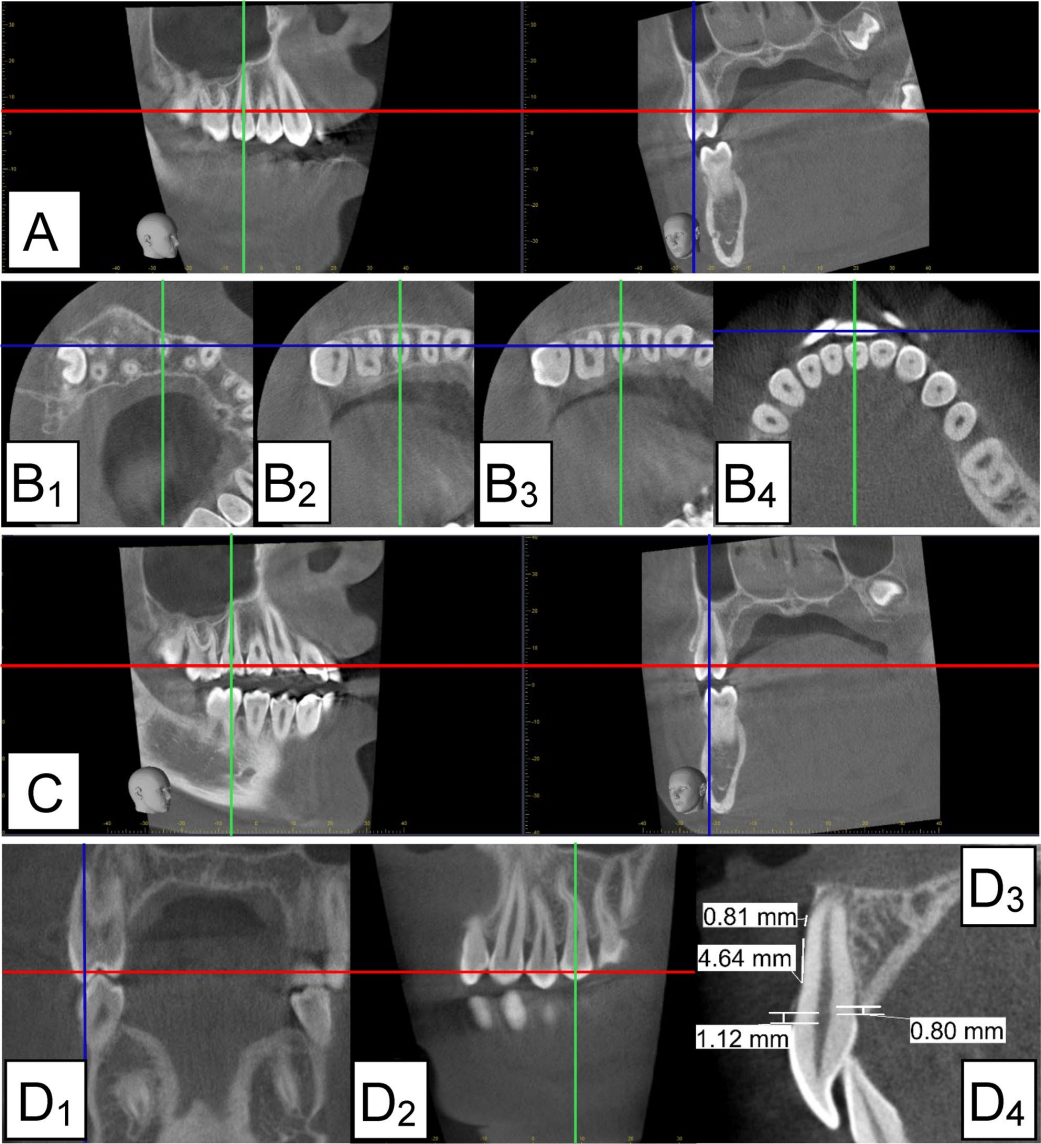
Measurement protocol. Primary setting: Reference lines were aligned from the incisal edge, canine cusp tip, or buccal cusp tip of premolars to the root apex (**A**). For multirooted teeth, the line extended from the buccal cusp tip to the buccal root apex. In the axial stack, the sagittal reference line was positioned to intersect mid-buccal and mid-palatal/lingual aspects of the root. This orientation was adjusted at several levels along the length of the root, starting at the apex, with final positioning approximately 2 mm below CEJ (**B**_**1**_–**B**_**3**_). For incisors, orientation was based on the incisal edge at the level of the dentinoenamel junction (**B**_**4**_). Final adjustments: In the sagittal and coronal stacks, axial reference lines were aligned with the long axis of the tooth. The long axis was defined as passing through the mid-incisal edge (incisors), cusp tip (canines), buccal cusp tip (two-rooted premolars), or mid central groove (single-rooted premolars), at the level of the dentinoenamel junction, and extending to the apical point of the root (**C**). For multirooted teeth, the buccal root was selected (**D**_**1**_). Curved root segments were ignored (**D**_**2**_). Measurements: In the sagittal stack, marker lines were placed at the CEJ and the alveolar bone crest, with positions verified by scrolling through the axial stack. Measurements were performed using the ruler function (**D**_**3**_). Fenestrations: Following linear measurements, the tooth was assessed for the presence of fenestrations (> 2.2 mm in vertical dimension) by scrolling through all planes (**D**_**4**_)

The measurement protocol (Fig. 1) was developed through interdisciplinary collaboration between an oral and maxillofacial radiologist (HC), two orthodontists (KJ, LP) experienced in CBCT research, and a general dentist (LBM), and was informed by previous research [14, 24]. The protocol was designed for assessment of alveolar bone by identifying anatomical buccal and palatal/lingual measurement sites for linear measurement of marginal bone level from the cementoenamel junction (CEJ) to the marginal bone crest in CBCT volumes, enabling longitudinal comparisons such as before and after treatment. Measurements were recorded in millimetres (mm), and fenestrations > 2.2 mm (measured vertically) were registered [14].

The protocol was discussed and finalised after the raters reached consensus. To ensure consistent application and software use, the raters reviewed all the procedural steps and performed calibrations using CBCT volumes not included in the study.

For each tooth, the XYZ planes were adjusted, and measurements were performed independently by the raters according to the protocol on pseudonymised CBCT volumes. Buccal and palatal/lingual measurements of second premolars, canines, and central incisors in the first and third quadrants were included to represent anterior and posterior regions of the maxillary and mandibular alveolar bone, encompassing areas with varying proportions of cortical and cancellous bone. Both T0 and T1 examinations were assessed to account for the potential effects of orthodontic treatment on bone density and, consequently, on agreement and reliability.

Three calibrated raters, KJ and HC (with more than 20 and 8 years of experience as specialists in orthodontics and maxillofacial radiology, respectively) and LBM (with more than 12 years of experience as a general dentist), measured the buccal and palatal/lingual marginal bone levels at T0 and T1 for interrater reliability assessment. Two raters (HC, LBM) registered fenestrations at T0 and T1 for interrater agreement. The least experienced rater (LBM) repeated all measurements and registrations after a minimum interval of four weeks to assess intrarater reliability and agreement, assuming that more experienced raters would achieve equal or higher reliability and agreement.

### Sample size calculation

A sample size of 48 subjects is recommended to achieve an ICC of 0.80 with a 95% confidence interval (CI) width of 0.20 and 80% probability, using three raters [28]. This sample size also allows estimation of random error using Dahlberg’s formula, which requires a minimum of 25–30 subjects [29].

### Statistical analysis

Statistical analyses were performed using IBM SPSS Statistics (version 31.0.1.0(49), Armonk, NY, USA), inspired by a previous CROWDIT study [30] and in consultation with a statistician.

#### Linear bone-level measurements

Intra- and interrater reliability were assessed using intraclass correlation coefficients (ICC), two-way random effects model, absolute agreement, single measurement/rater, with 95% CI [31, 32]. Analyses were performed for each measurement site (buccally and palatally/lingually) on upper and lower second premolars, canines, and central incisors, at each time point (T0 and T1). To enable comparisons with prior research, ICC estimates were calculated for pooled buccal and pooled palatal/lingual measurements. To provide descriptive information for the different CBCT units, all measurement sites were pooled at T0 and T1.

ICC estimates were interpreted according to general guidelines: < 0.50 = poor reliability; 0.50–0.75 = moderate reliability; 0.75–0.90 = good reliability; and > 0.90 = excellent reliability [32].

Intrarater agreement was visualised and evaluated using Bland‒Altman plots with 95% limits of agreement (LoA) [33] for maxillary and mandibular pooled buccal and pooled palatal/lingual measurements at T0 and T1, respectively. Systematic error was controlled with paired samples t-test per measurement site and time point.

Intra- and interrater agreement were assessed as random errors using Dahlberg’s formula (1) [34] for each measurement site, tooth, and time point. For comparison with previous studies, Dahlberg’s error was also calculated for pooled buccal and pooled palatal/lingual measurements at each time point.

1 $$\mathrm{D}\mathrm{a}\mathrm{h}\mathrm{l}\mathrm{b}\mathrm{e}\mathrm{r}\mathrm{g}^{\prime}\mathrm{s}\, \mathrm{r}\mathrm{a}\mathrm{n}\mathrm{d}\mathrm{o}\mathrm{m} \mathrm{e}\mathrm{r}\mathrm{r}\mathrm{o}\mathrm{r}=\sqrt{\frac{\sum {d}^{2}}{2\mathrm{n}}}$$

Standard error of measurement (SEM) (2) [23] was calculated for each measurement site, tooth and time point, using the mean square error (MS_E_) from the intrarater ICC ANOVA output. The minimal detectable change (MDC) with 95% confidence (MDC_95_) was calculated for each measurement site (3) [23].

2 $$\mathrm{S}\mathrm{E}\mathrm{M} =\sqrt{{MS}_{E}}$$

3 $$\mathrm{M}\mathrm{D}\mathrm{C}_{95} =1.96\times \surd 2\times SEM$$

#### Registration of fenestrations

Intra- and interrater agreement were assessed as percentage agreement.

#### Distribution and significance

Normality was assessed using Shapiro‒Wilk test and Q‒Q plots. Statistical significance was set at *P* < 0.05.

## Results

A total of 65 eligible participants were identified, and based on the sample size calculation, 48 were randomly selected for inclusion (20♀, 28♂) with a mean age of 14.4 years and a standard deviation (SD) of 1.79, yielding 96 available CBCT volumes. The number of CBCTs acquired using each CBCT unit and acquisition protocol is presented in Table 1. After random selection, one T0 scan was excluded due to extensive motion artefacts. Additionally, one upper and one lower second premolar at T0 were under eruption and were therefore excluded from analysis. In total, 1136 measurement sites were assessed, yielding 4544 linear measurements and 3408 fenestration assessments.

### Linear bone-level measurements

The intrarater ICCs at T0 and T1 ranged from moderate to excellent across all measurement sites. For pooled buccal measurements, the ICCs were 0.84 (95% CI 0.80–0.87) at T0 and 0.91 (95% CI 0.89–0.93) at T1; the corresponding palatal/lingual values were 0.81 (95% CI 0.77–0.85) and 0.86 (95% CI 0.82–0.88) (Table 2).

**Table 2:** Intrarater ICC and mean (mm) values for initial and repeated measurements at T0 and T1

		Mean (SD)			95% CI	
Measurement	n	Initial measurement	Repeated measurement	ICC	Lower	Upper
15b T0	46	0.93 (0.55)	0.99 (0.64)	0.716	0.541	0.832
15p T0	46	1.09 (0.45)	1.09 (0.45)	0.669	0.471	0.803
13b T0	47	1.64 (0.79)	1.65 (0.87)	0.821	0.699	0.896
13p T0	47	1.06 (0.62)	1.08 (0.67)	0.678	0.486	0.807
11b T0	47	1.43 (0.50)	1.41 (0.54)	0.803	0.671	0.885
11p T0	47	1.01 (0.54)	0.98 (0.47)	0.858	0.759	0.918
35b T0	46	0.91 (0.97)	0.89 (0.92)	0.894	0.816	0.940
35l T0	46	1.11 (0.49)	1.06 (0.53)	0.788	0.648	0.877
33b T0	47	1.57 (1.38)	1.78 (1.82)	0.807	0.679	0.887
33l T0	47	1.12 (0.71)	1.18 (0.84)	0.639	0.434	0.782
31b T0	47	1.72 (1.59)	1.65 (1.48)	0.849	0.744	0.913
31l T0	47	1.95 (1.08)	1.93 (1.03)	0.881	0.796	0.932
Pooled buccal sites at T0		1.37 (1.09)	1.40 (1.18)	0.840	0.802	0.871
Pooled lingual sites at T0		1.22 (0.75)	1.22 (0.77)	0.813	0.769	0.849
15b T1	48	1.02 (0.64)	1.10 (0.61)	0.571	0.346	0.734
15p T1	48	1.26 (0.45)	1.37 (0.56)	0.727	0.555	0.838
13b T1	48	2.84 (2.09)	2.79 (1.89)	0.709	0.533	0.826
13p T1	48	1.76 (1.14)	1.82 (1.04)	0.793	0.658	0.878
11b T1	48	1.76 (0.74)	1.83 (0.78)	0.937	0.889	0.964
11p T1	48	1.71 (1.19)	1.87 (1.18)	0.919	0.852	0.956
35b T1	48	1.25 (0.88)	1.33 (1.01)	0.770	0.624	0.864
35l T1	48	1.32 (0.56)	1.33 (0.67)	0.784	0.644	0.873
33b T1	48	2.53 (2.19)	2.46 (2.11)	0.950	0.913	0.972
33l T1	48	1.69 (1.08)	1.65 (0.96)	0.900	0.828	0.942
31b T1	48	4.19 (2.77)	4.34 (2.73)	0.943	0.901	0.968
31l T1	48	2.60 (1.85)	2.56 (1.41)	0.806	0.678	0.887
Pooled buccal sites at T1		2.26 (2.05)	2.31 (2.01)	0.910	0.888	0.928
Pooled lingual sites at T1		1.72 (1.21)	1.77 (1.08)	0.856	0.822	0.884

*ICC* intraclass correlation coefficient (two-way random effects model, absolute agreement, single measurement); *SD* standard deviation; *n* number of sites included in the analysis; *CI* confidence interval; *T0* baseline; *T1* post-active treatment

Intrarater ICC ranged from moderate to excellent, with 95% CI ranging from 0.346 to 0.972. F-test *P*-values were < 0.001 for all ICC estimates

Interrater ICCs ranged from poor to good. For pooled buccal measurements, ICCs were 0.60 (95% CI 0.54–0.66) at T0 and 0.73 (95% CI 0.62–0.81) at T1; the corresponding palatal/lingual values were 0.66 (95% CI 0.60–0.71) and 0.68 (95% CI 0.50–0.79) (Table 3).

**Table 3:** Interrater ICCs and mean (mm) values for measurements at T0 and T1 by rater (A, B, C)

		Mean (SD)				95% CI	
Measurement	n	Rater A	Rater B	Rater C	ICC	Lower	Upper
15b T0	46	0.93 (0.55)	1.01 (0.56)	0.79 (0.56)	0.679	0.531	0.796
15p T0	46	1.09 (0.45)	1.16 (0.42)	1.01 (0.38)	0.486	0.313	0.648
13b T0	47	1.64 (0.79)	2.37 (1.59)	1.86 (0.95)	0.327	0.151	0.511
13p T0	47	1.06 (0.62)	1.16 (0.61)	0.96 (0.66)	0.513	0.344	0.667
11b T0	47	1.43 (0.50)	1.64 (0.54)	1.49 (0.55)	0.644	0.490	0.769
11p T0	47	1.01 (0.54)	1.21 (0.55)	1.05 (0.47)	0.345	0.168	0.527
35b T0	46	0.91 (0.97)	0.84 (0.82)	0.93 (1.68)	0.407	0.224	0.585
35l T0	46	1.11 (0.49)	1.22 (0.45)	0.96 (0.45)	0.440	0.260	0.612
33b T0	47	1.57 (1.38)	1.89 (1.87)	2.27 (2.20)	0.622	0.468	0.752
33l T0	47	1.12 (0.71)	1.18 (0.54)	1.35 (0.86)	0.501	0.331	0.658
31b T0	47	1.72 (1.59)	2.09 (1.73)	2.30 (2.20)	0.625	0.474	0.753
31l T0	47	1.95 (1.08)	2.23 (1.22)	2.28 (1.81)	0.667	0.526	0.783
Pooled buccal sites at T0		1.37 (1.09)	1.64 (1.41)	1.61 (1.63)	0.600	0.537	0.658
Pooled lingual sites at T0		1.22 (0.75)	1.36 (0.79)	1.27 (1.03)	0.660	0.604	0.711
15b T1	48	1.02 (0.64)	1.01 (0.61)	1.05 (0.56)	0.638	0.490	0.762
15p T1	48	1.26 (0.45)	1.20 (0.41)	1.76 (0.65)	0.409	0.136	0.627
13b T1	48	2.84 (2.09)	3.14 (2.40)	4.52 (3.69)	0.488	0.303	0.653
13p T1	48	1.76 (1.14)	1.89 (1.03)	2.69 (1.02)	0.536	0.260	0.725
11b T1	48	1.76 (0.74)	1.77 (0.72)	2.44 (0.97)	0.657	0.274	0.833
11p T1	48	1.71 (1.19)	1.84 (1.10)	2.50 (1.33)	0.753	0.465	0.879
35b T1	48	1.25 (0.88)	1.10 (0.88)	1.89 (1.71)	0.400	0.217	0.577
35l T1	48	1.32 (0.56)	1.41 (0.45)	1.87 (0.88)	0.324	0.136	0.513
33b T1	48	2.53 (2.19)	2.51 (2.40)	3.80 (3.05)	0.745	0.551	0.856
33l T1	48	1.69 (1.08)	1.59 (0.87)	2.63 (1.53)	0.623	0.270	0.806
31b T1	48	4.19 (2.77)	4.14 (2.74)	6.15 (2.85)	0.730	0.360	0.875
31l T1	48	2.60 (1.85)	2.86 (2.12)	3.82 (2.50)	0.669	0.482	0.798
Pooled buccal sites at T1		2.26 (2.05)	2.28 (2.17)	3.31 (2.95)	0.729	0.616	0.805
Pooled lingual sites at T1		1.72 (1.21)	1.80 (1.26)	2.54 (1.59)	0.680	0.495	0.789

*ICC* intraclass correlation coefficient (two-way random effects model, absolute agreement, single rater); *SD* standard deviation; *n* number of sites included in the analysis; *CI* confidence interval; *T0* baseline; *T1* post-active treatment

Interrater ICC ranged from poor to good, with 95% CI ranging from 0.136 to 0.879. F-test *P*-values were < 0.001 for all ICC estimates

For the different CBCT units, intrarater ICCs ranged from good to excellent at both T0 and T1, with one moderate estimate, whereas interrater ICCs ranged from moderate to good, with one poor estimate (Table 4).

**Table 4:** Intrarater and interrater ICCs and means (mm) at T0 and T1 by CBCT-unit and rater

CBCT units at T0	Clinic	Intrarater reliability					
		n	Rater A		ICC	95% CI	
			Rater A1 mean (SD)	Rater A2 mean (SD)		Lower	Upper
All CBCT units	ABC	560	1.29 (0.94)	1.31 (1.00)	0.833	0.805	0.856
3D Accuitomo^®^ 170	A	108	1.38 (0.68)	1.44 (0.74)	0.733	0.632	0.809
Veraviewepocs^®^	A	156	1.54 (1.09)	1.60 (1.24)	0.819	0.760	0.865
i-CAT^®^ 9140	B	236	1.12 (0.93)	1.07 (0.92)	0.851	0.811	0.882
3D Accuitomo^®^ 170	C	60	1.19 (0.77)	1.29 (0.77)	0.870	0.789	0.921
CBCT units at T1							
All CBCT units	ABC	576	1.99 (1.71)	2.04 (1.63)	0.900	0.883	0.914
Veraviewepocs^®^	A	276	2.32 (1.81)	2.29 (1.71)	0.878	0.848	0.902
i-CAT^®^ 9140	B	84	1.87 (1.89)	1.99 (1.81)	0.849	0.777	0.899
ProMax^®^ 3D Mid	B	156	1.46 (1.18)	1.59 (1.20)	0.961	0.935	0.975
3D Accuitomo^®^ 170	C	60	2.04 (1.76)	2.07 (1.77)	0.974	0.956	0.984

CBCT units at T0	Clinic	Interrater reliability						
		n	Raters (A, B, C)			ICC	95% CI	
			Rater A1 mean (SD)	Rater B mean (SD)	Rater C mean (SD)		Lower	Upper
All CBCT units	ABC	560	1.29 (0.94)	1.44 (1.37)	1.50 (1.15)	0.620	0.578	0.661
3D Accuitomo^®^ 170	A	108	1.38 (0.68)	1.27 (1.09)	1.60 (1.41)	0.439	0.323	0.552
Veraviewepocs^®^	A	156	1.54 (1.09)	1.74 (1.81)	1.48 (1.33)	0.704	0.634	0.765
i-CAT^®^ 9140	B	236	1.12 (0.93)	1.30 (1.19)	1.54 (0.92)	0.576	0.491	0.651
3D Accuitomo^®^ 170	C	60	1.19 (0.77)	1.54 (1.01)	1.23 (0.94)	0.776	0.651	0.860
CBCT units at T1								
All CBCT units	ABC	576	1.99 (1.71)	2.93 (2.40)	2.04 (1.78)	0.722	0.599	0.801
Veraviewepocs^®^	A	276	2.32 (1.81)	3.05 (2.44)	2.16 (1.81)	0.770	0.660	0.839
i-CAT^®^ 9140	B	84	1.87 (1.89)	3.59 (3.22)	2.31 (2.06)	0.603	0.401	0.741
ProMax^®^ 3D Mid	B	156	1.46 (1.18)	2.52 (1.82)	1.67 (1.39)	0.649	0.423	0.779
3D Accuitomo^®^ 170	C	60	2.04 (1.76)	2.47 (1.93)	2.05 (2.03)	0.863	0.791	0.913

*ICC* intraclass correlation coefficient (two-way random effects model, absolute agreement, single measurement/rater); *SD* standard deviation; *n* number of measured sites; *Rater A1* rater A observation 1; *Rater A2* rater A observation 2; *CBCT* cone-beam computed tomography; *CI* confidence interval; *T0* baseline; *T1*, post-active treatment; Clinic, radiology clinic (A-C)

Intrarater ICC ranged from moderate to excellent, and interrater ICC ranged from poor to good. F-test *P*-values were < 0.001 for all ICC estimates

**Fig. 2:**
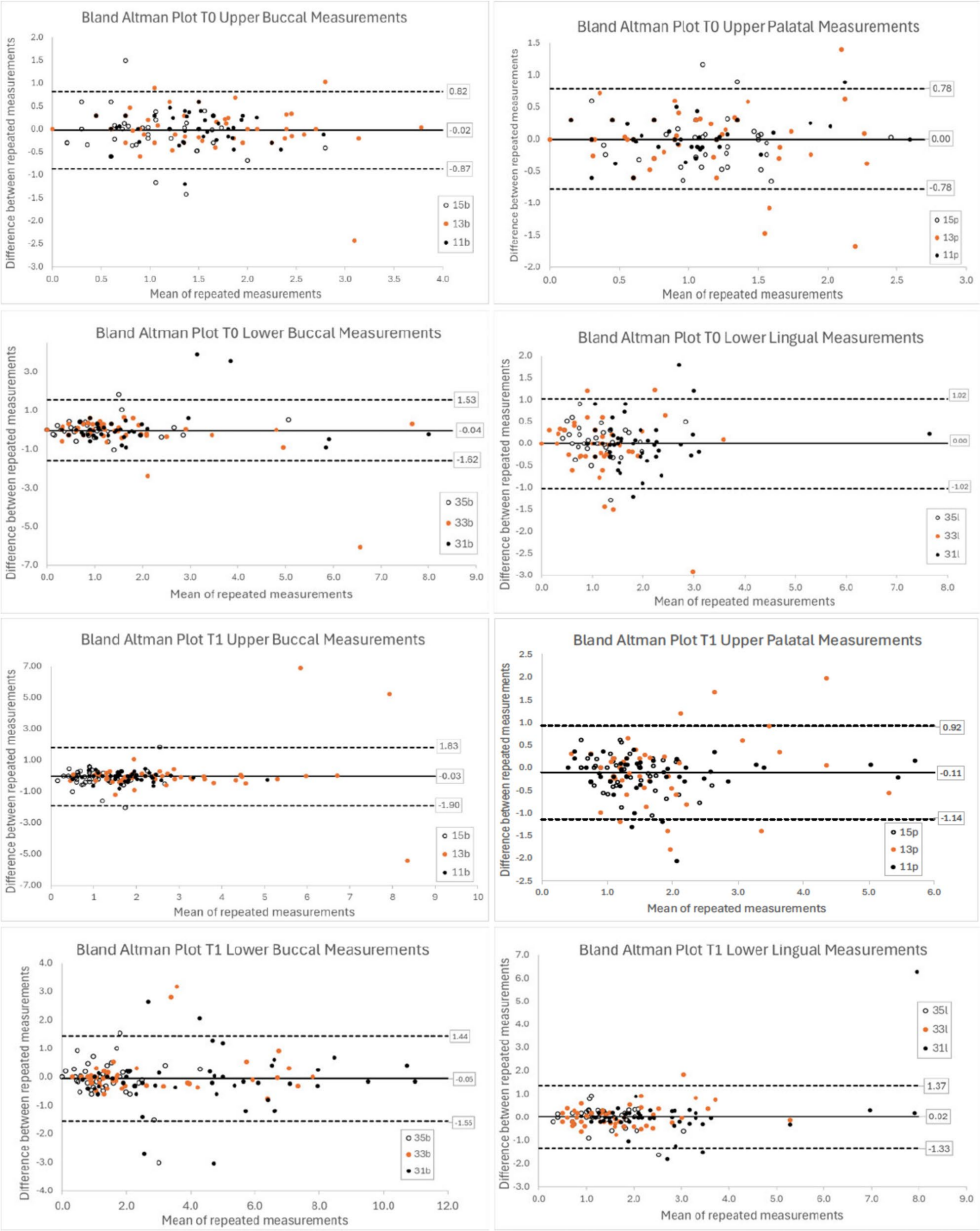
Bland–Altman plots for repeated measurements of marginal bone level at T0 and T1. *T0* baseline; *T1* post-active treatment; mm, millimetres. ≥ 95% of the differences were within the LoA, except for lower lingual measurements at T0 (94%), upper palatal measurements at T1 (93%) and lower buccal measurements at T1 (94%)

The Bland–Altman plots indicated minimal systematic bias, with mean differences close to zero (Fig. 2). Paired samples t-tests revealed statistically significant mean differences for 15p at T1 (− 0.11 mm; *P* = 0.037) and 11p at T1 (− 0.16 mm; *P* = 0.016). The LoA varied by site and time point, with the narrowest range observed for maxillary measurements at T0. Additional plots for each tooth and measurement site are provided in Additional file 1. Sensitivity analyses excluding outliers [35] (> 3 SD) yielded narrower LoA, ranging from − 0.89 to 0.86 mm at T0, and from − 1.01 to 0.90 mm at T1 (Additional file 2).

Intrarater random error ranged from 0.19 to 0.71 mm at T0 and from 0.19 to 0.72 mm at T1, except at site 13b (1.07 mm, T1). Interrater random error ranged from 0.27 to 1.27 mm at T0 and from 0.23 to 1.87 mm at T1, with higher values observed at site 13b (up to 2.54 mm) (Table 5).

**Table 5:** Intrarater and interrater random error (mm) at T0 and T1

Site		Intrarater				Interrater			
		Rater A1 and A2		Rater A and B		Rater A and C		Rater B and C	
		n	Dahlberg	n	Dahlberg	n	Dahlberg	n	Dahlberg
T0	15b	46	0.32	46	0.34	46	0.27	46	0.34
	15p	46	0.26	46	0.28	46	0.34	46	0.28
	13b	47	0.35	47	0.42	47	1.19	47	1.16
	13p	47	0.37	47	0.39	47	0.51	47	0.42
	11b	47	0.23	47	0.28	47	0.32	47	0.37
	11p	47	0.19	47	0.40	47	0.47	47	0.41
	35b	46	0.31	46	1.07	46	0.48	46	1.10
	35 l	46	0.23	46	0.32	46	0.32	46	0.42
	33b	47	0.71	47	1.24	47	1.22	47	0.96
	33 l	47	0.47	47	0.49	47	0.45	47	0.57
	31b	47	0.60	47	1.27	47	0.91	47	1.22
	31 l	47	0.36	47	0.84	47	0.52	47	0.99
T1	15b	48	0.41	48	0.43	48	0.32	48	0.33
	15p	48	0.27	48	0.53	48	0.23	48	0.55
	13b	48	1.07	48	2.45	48	0.92	48	2.54
	13p	48	0.50	48	0.95	48	0.69	48	0.74
	11b	48	0.19	48	0.62	48	0.33	48	0.60
	11p	48	0.34	48	0.72	48	0.51	48	0.66
	35b	48	0.46	48	1.12	48	0.52	48	1.20
	35 l	48	0.28	48	0.71	48	0.28	48	0.68
	33b	48	0.48	48	1.59	48	0.70	48	1.57
	33 l	48	0.32	48	0.90	48	0.48	48	0.97
	31b	48	0.65	48	1.86	48	0.64	48	1.87
	31 l	48	0.72	48	1.60	48	1.06	48	1.19

For pooled measurements, the random errors were 0.45 mm (T0) and 0.61 mm (T1) buccally, and 0.33 mm (T0) and 0.44 mm (T1) palatally/lingually. SEM ranged from 0.19 to 1.08 mm, and MDC_95_ ranged from 0.53 to 1.94 mm (Table 6).

**Table 6:** Standard error of measurement (SEM) at T0 and T1, and minimal detectable change (MDC_95_) from T0 to T1, by site (rater A). All values in mm

Site	n	SEM T0	SEM T1	MDC_95_
15b	46	0.32	0.41	0.89
15p	46	0.26	0.26	0.72
13b	47	0.35	1.08	0.97
13p	47	0.37	0.50	1.03
11b	47	0.23	0.19	0.64
11p	47	0.19	0.32	0.53
35b	46	0.31	0.46	0.86
35 l	46	0.23	0.29	0.64
33b	47	0.70	0.48	1.94
33 l	47	0.47	0.33	1.30
31b	47	0.60	0.65	1.66
31 l	47	0.37	0.73	1.03

Some variables deviated from normality according to the Shapiro‒Wilk test, although Q‒Q plots indicated approximate normality, with deviations mainly in the tails due to outliers.

### Fenestrations

**Fig. 3:**
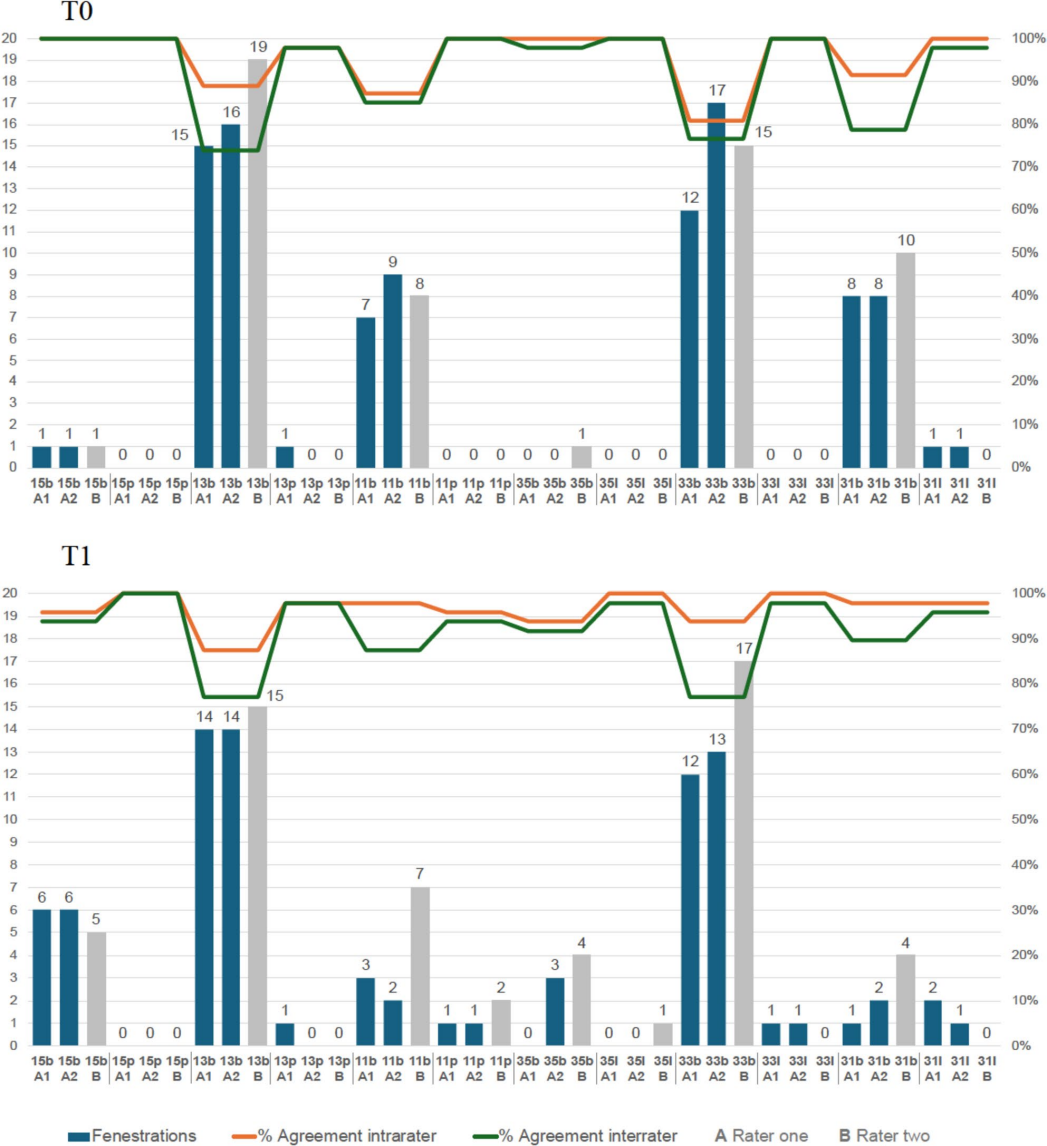
Number of fenestrations (bars, left y-axis) identified by Rater A (initial and repeated assessments) and Rater B, and corresponding intrarater and interrater percentage agreement (lines, right y-axis) at T0 and T1. *T0* baseline; *T1* post-active treatment

The intrarater and interrater percentage agreements were high, exceeding 81% and 74%, respectively (Fig. 3). All contingency tables are provided in Additional file 3.

## Discussion

The findings of this study indicate that the measurement protocol is suitable for assessing marginal bone levels before and after orthodontic treatment in adolescents when measurements are performed by the same rater. Agreement for the detection of fenestrations was high, exceeding 81% for the intrarater and 74% for the interrater assessments. Given the recognised need for long-term follow-up research of orthodontic treatment in children [36], the present study contributes to future investigations on alveolar bone changes over time by providing a well-defined and thoroughly evaluated measurement protocol.

Although CBCT is not recommended for routine evaluation in orthodontic everyday practice because of higher radiation exposure than 2D imaging, it is justified for specific clinical indications and remains valuable for research purposes [37]. According to ALADAIP (as low as diagnostically acceptable, being indication-oriented and patient-specific), image quality should be balanced against the radiation dose and tailored to the diagnostic task and patient [16]. Accordingly, reliability and agreement studies are essential and should be conducted in accordance with published recommendations [26].

### Measurement protocols

Measurement protocols vary between studies depending on their specific purposes [3, 24], influencing both the selection of measurement sites and the outcomes. Previous reliability and agreement studies in CBCT have been conducted in vitro [18], ex vivo [3], and using preselected (static) images [5]. The latter, commonly used in interobserver variability research in diagnostic imaging to assess image interpretation [38], can be appropriate for specific aims [39]. However, it does not capture the full diagnostic process and may therefore yield optimistically high estimates of reliability and agreement.

In clinical follow-up studies, measurability in CBCT images may be affected by factors influencing landmark visibility and image quality, such as orthodontic treatment-related changes [25], patient positioning [40], and movement during acquisition [41]. Given scan times of up to 17.5 s in the present study, some patient movement is expected, particularly in young individuals [12], potentially degrading image quality through distortion artefacts [42]. In addition, the raters must select image slices within the XYZ MPR stacks, adjust the image orientation, and consistently identify the same anatomical landmarks across different time points, further challenging reliability. Thus, in clinically relevant reliability studies, the entire measurement process should be repeated, rather than relying solely on repeated assessments of preselected static images, because variability introduced during image selection, orientation, and landmark identification would otherwise go unmeasured [38, 43].

When curvilinear MPR is used to orient reference lines along the dental arch, buccal bone measurements in rotated teeth may inadvertently be taken adjacent to mesial or distal surfaces. The CEJ, a common reference for marginal bone level assessment, is not uniformly positioned but varies circumferentially around the tooth, increasing the risk of measurement error, particularly in orthodontic follow-up studies where tooth movement and derotation may result in measurements being taken at different sites and CEJ levels compared with baseline. Conversely, orienting CBCT reference lines according to tooth anatomy, as in the present study, may lead to palatal/lingual measurements inadvertently being taken at the interproximal bone in rotated teeth.

Maintaining consistent axial plane orientation across CBCT volumes is also challenging, especially in teeth with circular root morphology. In the present study, the full root length was used to align the raters’ views, with axial orientation initiated at the apex and finalised approximately two millimetres below the CEJ.

Coronal anatomical landmarks proposed in earlier studies, such as the incisal edge [3, 24], are susceptible to attrition and abrasion. Therefore, crown landmarks at the dentinoenamel junction were used to orient the reference lines in the present study, minimising the impact of such changes on the follow-up assessment.

### Patient characteristics

Pronounced crowding and contact point displacements at T0 were occasionally perceived to complicate assessments, as adjacent teeth could influence landmark detection. In addition, orthodontic treatment may alter alveolar bone density, particularly on the pressure side [25], potentially complicating detection of the marginal bone crest at T1, especially at the incisors, where the alveolar bone covering is predominantly thin [44].

### Intraclass correlation coefficients

In the present study, intrarater reliability for bone level measurements ranged from moderate to excellent and was generally higher than that reported in a previous in vivo study assessing marginal bone levels in ten adolescents with crowding, despite the use of preselected images in that study [5]. Their intrarater ICCs were 0.56–0.57 across three independent raters for pooled maxillary and mandibular marginal bone level measurements at proximal surfaces, including incisors to first molars. Their interrater ICC involving six raters was 0.40 (95% CI: 0.32–0.47), whereas their pairwise interrater ICCs ranged from 0.34 to 0.68 [5]. These values were comparable to the interrater ICCs observed in the present study, which ranged from 0.33 to 0.68 at T0 (Table 3), with a pooled ICC of 0.62 for all measurements at T0 (Table 4), despite the inclusion of three raters, different CBCT units, and no image preselection. The structured measurement protocol used in the present study may have contributed to the relatively higher ICCs observed, though reliability may vary between surfaces.

The measurement protocol included axial plane orientation according to the incisal edge of the incisors and scrolling through the axial stack to identify the anatomical landmarks, which facilitated slice selection, reference line adjustment, and measurement point identification according to the raters. A similar approach demonstrated excellent interrater reliability (ICC: 0.92, 95% CI: 0.81–0.96) in periodontally affected central incisors using artefact-free CBCT volumes and involving two raters [24]. Differences in ICC estimates may partly reflect differences in study populations, as ICC values are influenced by between-subject variability, with greater variability resulting in higher ICC values when measurement error remains constant [17]. In contrast, the inclusion criteria in the present study resulted in a homogeneous sample at T0, thereby limiting between-subject variability.

A dose-optimisation study evaluating 12 acquisition protocols with 8 × 8 FOV and 160 µm voxel size and varying exposure parameters reported high interrater reliability among five raters (ICC: 0.85 and 0.95 for buccal and palatal/lingual measurements, respectively) [21]. The use of a RANDO^®^ skull phantom and preselected images eliminated motion artefacts and image selection variability, which may partly explain the higher ICC values reported in that study [21]. As voxel size and FOV influence image quality, with larger voxel sizes reducing spatial resolution and larger FOVs increasing noise and artefacts [45], differences in these parameters may also have affected measurement variability. Despite this, intrarater reliability in the present study was good to excellent for pooled measurements from the different CBCT units and time points, with the exception of one moderate ICC (Table 4). However, unequal subgroup sizes and non-random allocation to CBCT units preclude comparative evaluation of CBCT units or acquisition protocols, which was not the aim of the present study. Therefore, the CBCT unit specific results should be interpreted as descriptive rather than comparative.

Although the ICCs indicated mostly good to excellent intrarater reliability and moderate to good interrater reliability, some confidence intervals extended into the poor range and should be considered when interpreting the results (Tables 2, 3 and 4). Overall, the findings support the use of the measurement protocol in follow-up studies when all assessments are performed by the same rater. Although averaging repeated measurements may further improve reliability by reducing random error [23, 43], this approach is more resource- and labour-intensive and was not applied in the present study.

### Bland–Altman plot

Bland–Altman plots display the differences between paired measurements against their means, systematic bias as estimated by the mean difference, and the LoA within which 95% of the differences are expected to lie when normally distributed [33, 35].

In the present study, the mean differences between repeated measurements at T0 and T1 were close to zero, indicating minimal systematic bias. Although statistically significant mean differences were observed at two sites, their magnitudes were small and did not remain significant after adjustment for multiple comparisons. These isolated findings may therefore reflect chance.

The LoA can be used to assess whether measurement error is clinically relevant [35, 46]. In this study, they reflected the distribution of differences across combined measurement sites. Approximately 95% of the differences fell within the LoA, although their width varied across sites and time points, and some outliers were observed.

The widest LoA were observed for upper buccal measurements at T1 (Fig. 2). When 13b was analysed separately (Additional file 1), the LoA were approximately ± 3 mm, although 94% of the observations fell within ± 1 mm. After exclusion of three outliers, the LoA narrowed to ± 0.67 mm, illustrating their influence. A sensitivity analysis excluding observations exceeding three SDs was therefore performed for the site specific Bland–Altman plots to evaluate the impact of outliers (Additional file 2). This reduced all LoA to between − 0.89 and 0.86 mm at T0 and − 1.01 to 0.90 mm at T1, indicating that the observed variability was largely driven by outliers and that precision was adequate for most measurements. However, exclusion of outliers is not recommended except when assessing their influence [35].

The outliers, particularly for incisors and canines, likely reflect difficulties in detecting thin alveolar bone, especially at T0 in patients with pronounced anterior irregularity and labially displaced canines. At T1, buccal alveolar bone may remain thin following expansion, further complicating visualisation and measurement. The buccal bone of mandibular anterior teeth must be approximately 0.173 mm wide, and twice as wide lingually, to be reliably visualised on CBCT using a high resolution protocol [13].

Hence, despite a detailed measurement protocol and calibration, landmark identification may remain challenging. In a clinical setting, repeated assessments and careful visual evaluation of the CBCT image may be valuable in challenging cases. Measurement variability may also arise from differences in slice selection within the CBCT volumes and from rater-dependent interpretations of anatomical structures, potentially resulting in discrepant assessments of bone levels and fenestrations.

A 4-week retest interval was used to minimise memory effects. However, learning effects during the observation period may have contributed to measurement error, as the rater performing the repeated measurements had limited prior CBCT experience and assessed a large sample. Nevertheless, only minor systematic errors were observed, suggesting minimal impact on the overall results.

### Dahlberg’s random error

Dahlberg’s formula is frequently used in orthodontic research to quantify random error in repeated measurements [34, 43, 47], and assumes the absence of systematic error [29].

A previous study of 13 adolescents treated for crowding with premolar extraction and fixed appliances reported random errors for buccal and palatal/lingual measurements (0.31 mm buccally and 0.21 mm palatally/lingually at baseline, and 0.29 mm and 0.25 mm after treatment, respectively) [4]. In the present study, random error varied across measurement sites (Table 5). When the sites were pooled in a comparable manner, the buccal and palatal/lingual random errors were higher (0.45 and 0.33 mm at T0, 0.61 and 0.44 mm at T1, respectively).

The higher random error may partly reflect acquisition parameters associated with lower image quality, potentially complicating landmark detection compared with the previous study (60 × 60 mm volume, 17.5 s, 360° rotation, 75 kV and 4–5.5 mA) [4].

### Standard error of measurement and minimal detectable change

For an observed change to be considered meaningful, it must exceed measurement uncertainty. This can be assessed using the MDC, which is the smallest change required to conclude that an observed change is unlikely attributable solely to measurement error [23]. MDC is derived from the SEM, which quantifies the expected error of a measurement obtained under consistent conditions. Both SEM and MDC are expressed in the same units as the original measurements and are inversely related to ICC [23].

In the present study, SEM was calculated from repeated intrarater measurements at each time point (Table 6) and therefore reflects measurement error when assessments are performed by the same rater. The clinical relevance of SEM and MDC values should be interpreted based on clinical judgement [23]. A threshold of 2.2 mm has been reported to provide the highest diagnostic accuracy for detecting dehiscences and fenestrations on CBCT when compared with clinical findings [14]. Although this threshold was established for diagnostic purposes, it provides a useful reference magnitude, as measurement error should be small relative to defect sizes considered relevant. All SEM values were below this threshold, and lower than the mean bone levels observed at the respective measurement sites (Tables 2 and 6), and lower than the mean bone levels reported in a previous study of adolescent orthodontic patients [2].

In follow-up studies assessing change, at least two measurements contribute to the total measurement error [17]. The MDC represents the minimum observed change required to exceed measurement error and is therefore particularly relevant when interpreting treatment-related changes.

The standard formula for MDC assumes equal SEM values at both time points. As shown in Table 6, SEM values at T0 and T1 were generally comparable, with some exceptions, most notably at site 13b, where the MDC may be underestimated. The three large outliers identified at 13b at T1 (Fig. 2, Additional file 2) may partly explain the higher SEM at T1 and likely reflect difficulties in identifying the alveolar bone crest in cases with thin and diffusely defined buccal bone, possibly following orthodontic expansion. The MDC results should be interpreted in the context of these considerations.

To facilitate clinical interpretation of the observed MDC values, they may be considered in relation to the treatment-related bone level changes reported in previous CBCT studies of adolescent orthodontic patients. One study found that 58–95% of buccal and palatal/lingual tooth surfaces had an increased CEJ to marginal bone level distance after treatment, depending on tooth and surface [2]. Among the patients with increased distances, all had at least one surface with an increase of > 1 mm [2]. Accordingly, the ability to detect a CBCT derived marginal bone level change of at least 1 mm may be clinically relevant in the present context.

Based on this threshold, detection of a CBCT-derived marginal bone level change of > 1 mm appears feasible at all sites except teeth 31, 33 and site 13p, where MDC_95_ exceeded 1 mm. However, larger treatment-related mean changes have been reported at these sites (e.g. 31l, 5.7 mm; 33l, 1.4 mm; > 2 mm in > 22% of buccal surfaces at 31 and 33; > 2 mm in 16.3% of 13 palatal surfaces) [2]. Taken together, these findings suggest that treatment-related bone-level changes of magnitudes previously reported in adolescent orthodontic patients can be measured and distinguished from measurement error using the present method under the described conditions.

### Percentage agreement

Errors related to fenestrations are reflected in the percentage agreement, which exceeded 74%, with most disagreements observed for canines and incisors (Fig. 3, Additional file 3).

Assessment of chance-corrected agreement using the kappa coefficient would have been informative for clinical interpretation. However, kappa requires variability, which was lacking for some variables in the present study. In addition, when prevalence is very high or low, kappa may be substantially reduced despite high percentage agreement (the “kappa paradox”) [17, 48]. Therefore, percentage agreement was used. This should be considered when interpreting the results, as high percentage agreement does not necessarily correspond to high kappa values, since agreement occurring by chance is not accounted for [17].

### Strengths, limitations, and future research

Multiple raters evaluated the MPR-based protocol using CBCT volumes from age-appropriate orthodontic patients recruited through a multicentre RCT, enhancing the clinical relevance of the findings. Reliability and agreement were assessed across sites with varying anatomical characteristics, at both T0 and T1. A priori sample size estimation ensured an adequately powered sample, representing a methodological strength compared with previous studies [38].

Prior to trial initiation, objective image quality was assessed using the SEDENTEXCT phantom, followed by necessary parameter adjustments to establish customised, dose-optimised protocols for each CBCT unit in accordance with the ALADA principle, while accounting for the technical capabilities and limitations of the available CBCT units. Nevertheless, residual differences in image quality remained according to the authors' subjective assessment. This variation may have contributed to measurement variability and could limit the generalisability of the findings. At the same time, this heterogeneity reflects a situation commonly encountered in both clinical practice and research, where patients may undergo CBCT examinations at different radiology clinics. A formal comparison of CBCT units and acquisition protocols was beyond the scope of the present study. Future studies may consider incorporating both objective and subjective image quality assessments, such as observer-based evaluation of anthropomorphic phantom images.

Furthermore, several technical and biological factors may affect the visualisation of thin alveolar bone in CBCT images [15]. Consequently, the absence of visible bone on CBCT does not necessarily indicate a complete absence of bone in vivo [15]. As the validity of CBCT-based assessments relative to actual clinical bone levels was not evaluated, the present findings should be interpreted as reflecting measurement reliability and agreement under the conditions investigated.

Intrarater reliability was not established for all raters, which is an important limitation [23]. Consistently high intrarater reliability across raters would further support the suitability of the protocol for longitudinal assessment when measurements are performed by the same rater.

Caution is advised when generalising reliability and agreement estimates, as these depend on sample characteristics and assessment conditions [1, 23].

## Conclusions

This study evaluated the reliability and agreement of an MPR-based protocol for measuring linear marginal bone levels and detecting fenestrations in CBCT examinations acquired using multiple imaging units in adolescent orthodontic patients at baseline and following active non-extraction treatment.

The protocol demonstrated moderate to excellent intrarater reliability and agreement sufficient for assessing clinically relevant changes in alveolar marginal bone levels and identifying fenestrations in follow-up studies of adolescents treated for crowding without extractions. However, the results were influenced by rater variability and imaging conditions. The protocol performed most consistently when repeated assessments were conducted by the same rater, whereas additional calibration and/or repeated measurements may be required when multiple raters are involved. Assessment of reliability and agreement under study-specific conditions is recommended when applying the protocol in new research settings. These findings highlight both the potential and limitations of CBCT-based alveolar bone assessments and may inform future research in this field.

## Supplementary Information

Below is the link to the electronic supplementary material.

Supplementary Material 1.

Supplementary Material 2.

Supplementary Material 3.

## Data Availability

The datasets used and/or analysed during the current study are available from the corresponding author upon reasonable request.

## Abbreviations

ALADA: As low as diagnostically acceptable
ALADAIP: As low as diagnostically acceptable, being indication-oriented and patient-specific
ANOVA: Analysis of variance
CBCT: Cone beam computed tomography
CEJ: Cementoenamel junction
CI: Confidence interval
CROWDIT: Crowded Displaced Teeth
FOV: Field of view
GRRAS: Guidelines for Reporting Reliability and Agreement Studies
ICC: Intraclass correlation coefficient
LoA: Limits of agreement
MDC_95_: Minimal detectable change (at 95% confidence)
MPR: Multiplanar reconstruction
RCT: Randomised controlled trial
SD: Standard deviation
SEM: Standard error of measurement
T0: Baseline
T1: Post-active treatment
