# Supplementary material for: Reliability and agreement of CBCT-based alveolar bone assessments for follow-up studies on adolescent orthodontic patients using multiplanar reconstruction and various CBCT units
Source: Prog Orthod. 2026 Jul 21;27:35. doi: 10.1186/s40510-026-00637-y (PMC13388868; doi:10.1186/s40510-026-00637-y)
Supplement: Supplementary file 1 — Supplementary Material 1. [file 40510_2026_637_MOESM1_ESM.pdf]

Additional file 1. Bland-Altman plots for intrarater agreement of repeated measurements (mm) of marginal bone levels by measurement site at T0 and T1

T0

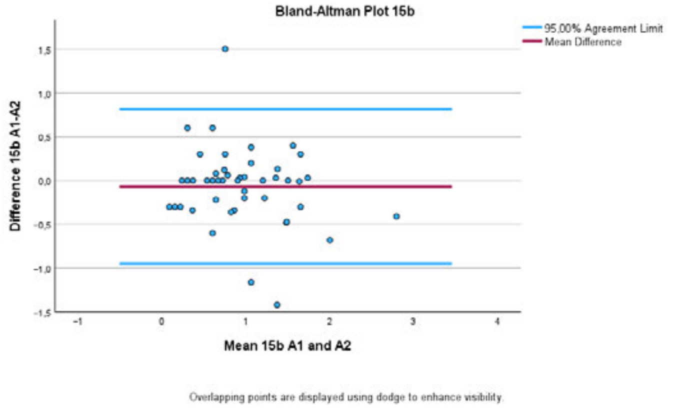

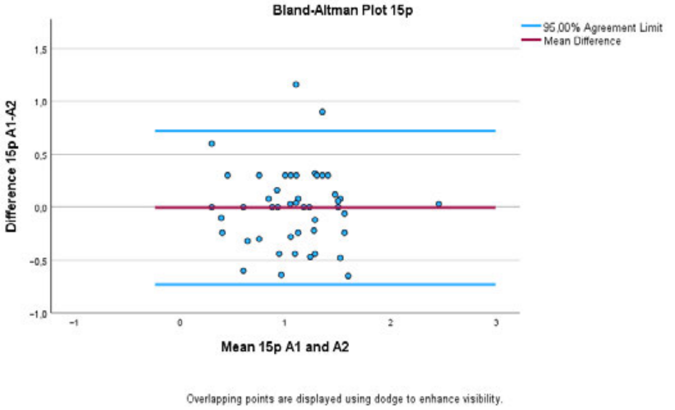

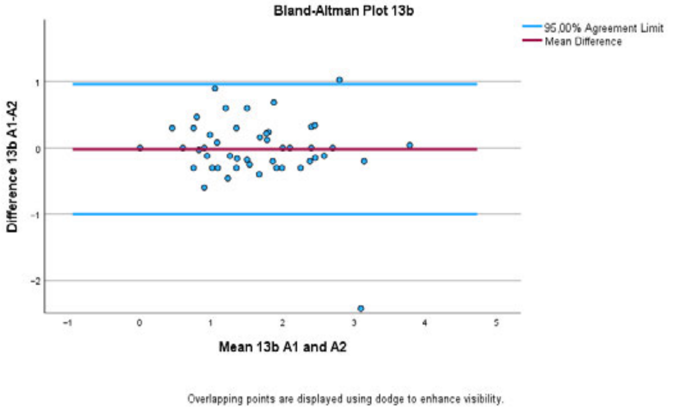

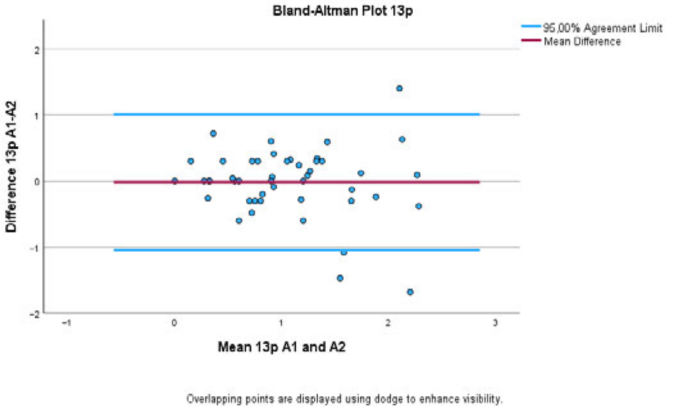

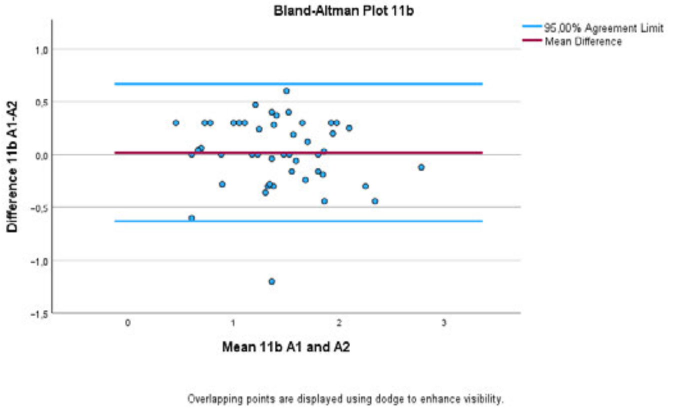

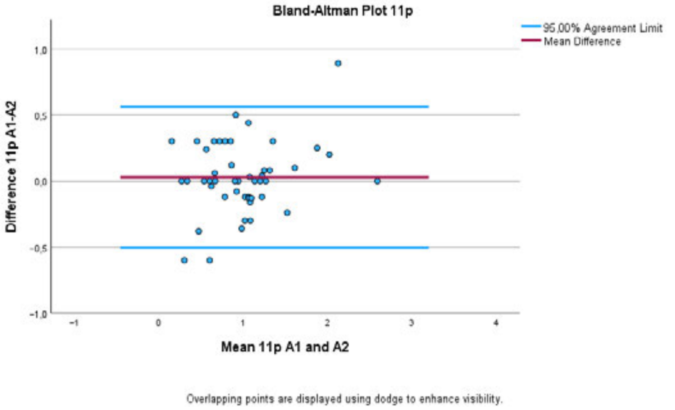

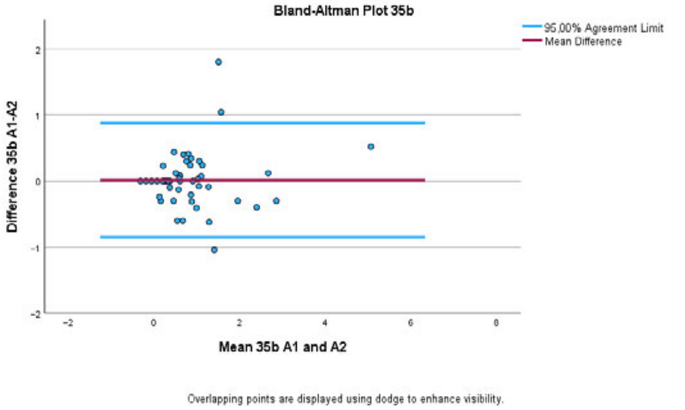

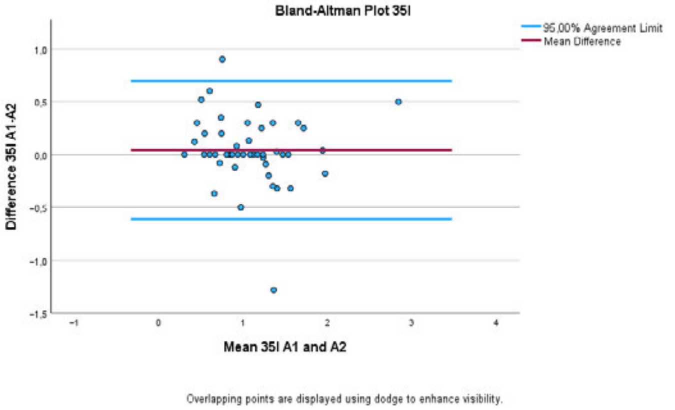

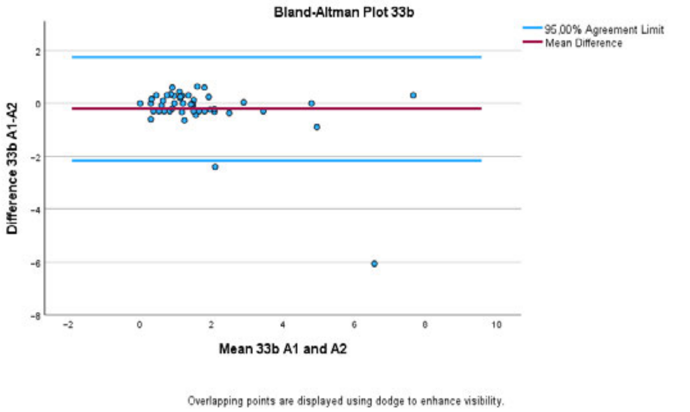
Bland-Altman Plot 33b
95,00% Agreement Limit
Mean Difference
Difference 33b A1-A2
Mean 33b A1 and A2
Overlapping points are displayed using dodge to enhance visibility.

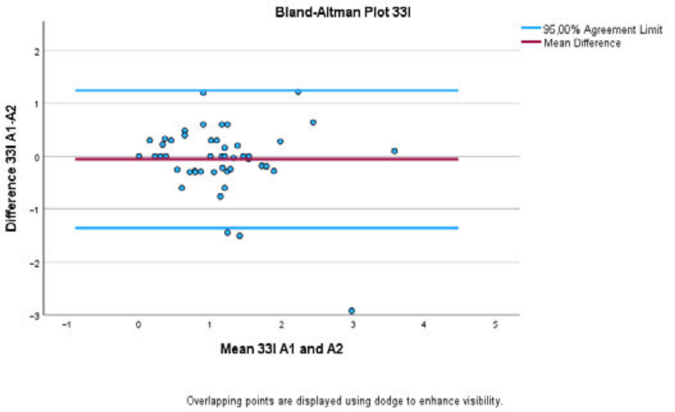
Bland-Altman Plot 33l
95,00% Agreement Limit
Mean Difference
Difference 33l A1-A2
Mean 33l A1 and A2
Overlapping points are displayed using dodge to enhance visibility.

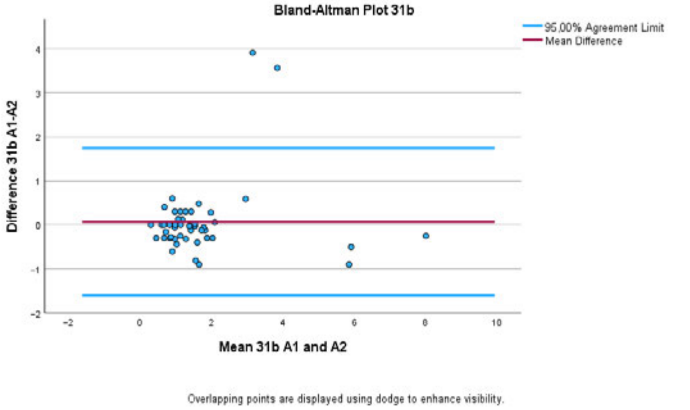
Bland-Altman Plot 31b
95,00% Agreement Limit
Mean Difference
Difference 31b A1-A2
Mean 31b A1 and A2
Overlapping points are displayed using dodge to enhance visibility.

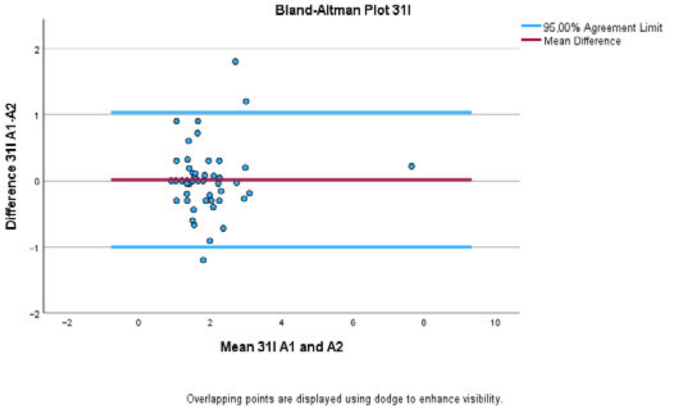
Bland-Altman Plot 31l
95,00% Agreement Limit
Mean Difference
Difference 31l A1-A2
Mean 31l A1 and A2
Overlapping points are displayed using dodge to enhance visibility.

T1

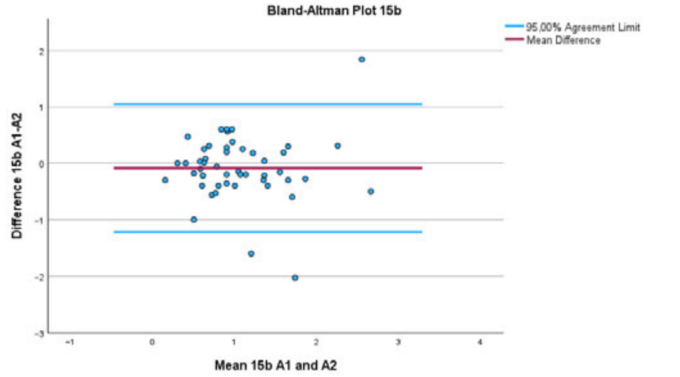
Bland-Altman Plot 15b
95,00% Agreement Limit
Mean Difference
Difference 15b A1-A2
Mean 15b A1 and A2

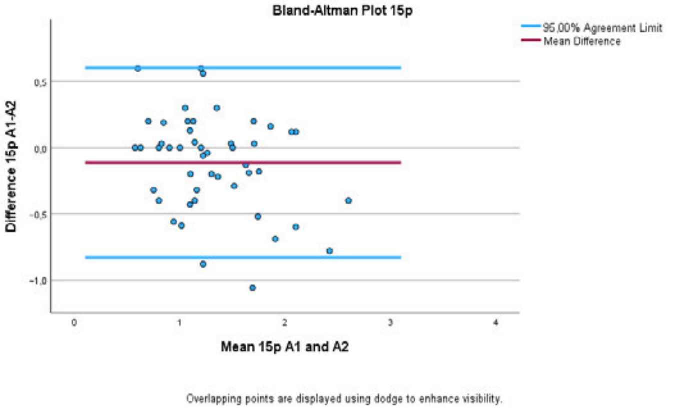
Bland-Altman Plot 15p
95,00% Agreement Limit
Mean Difference
Difference 15p A1-A2
Mean 15p A1 and A2
Overlapping points are displayed using dodge to enhance visibility.

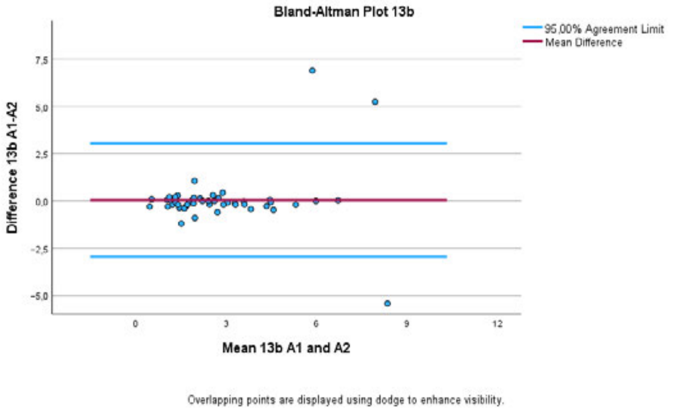
Bland-Altman Plot 13b
95,00% Agreement Limit
Mean Difference
Difference 13b A1-A2
Mean 13b A1 and A2
Overlapping points are displayed using dodge to enhance visibility.

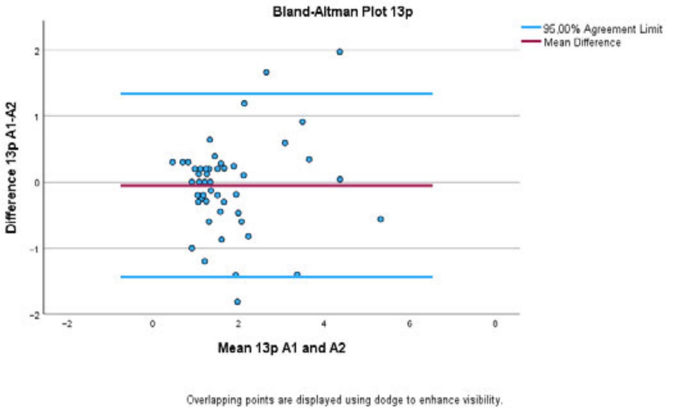
Bland-Altman Plot 13p
95,00% Agreement Limit
Mean Difference
Difference 13p A1-A2
Mean 13p A1 and A2
Overlapping points are displayed using dodge to enhance visibility.

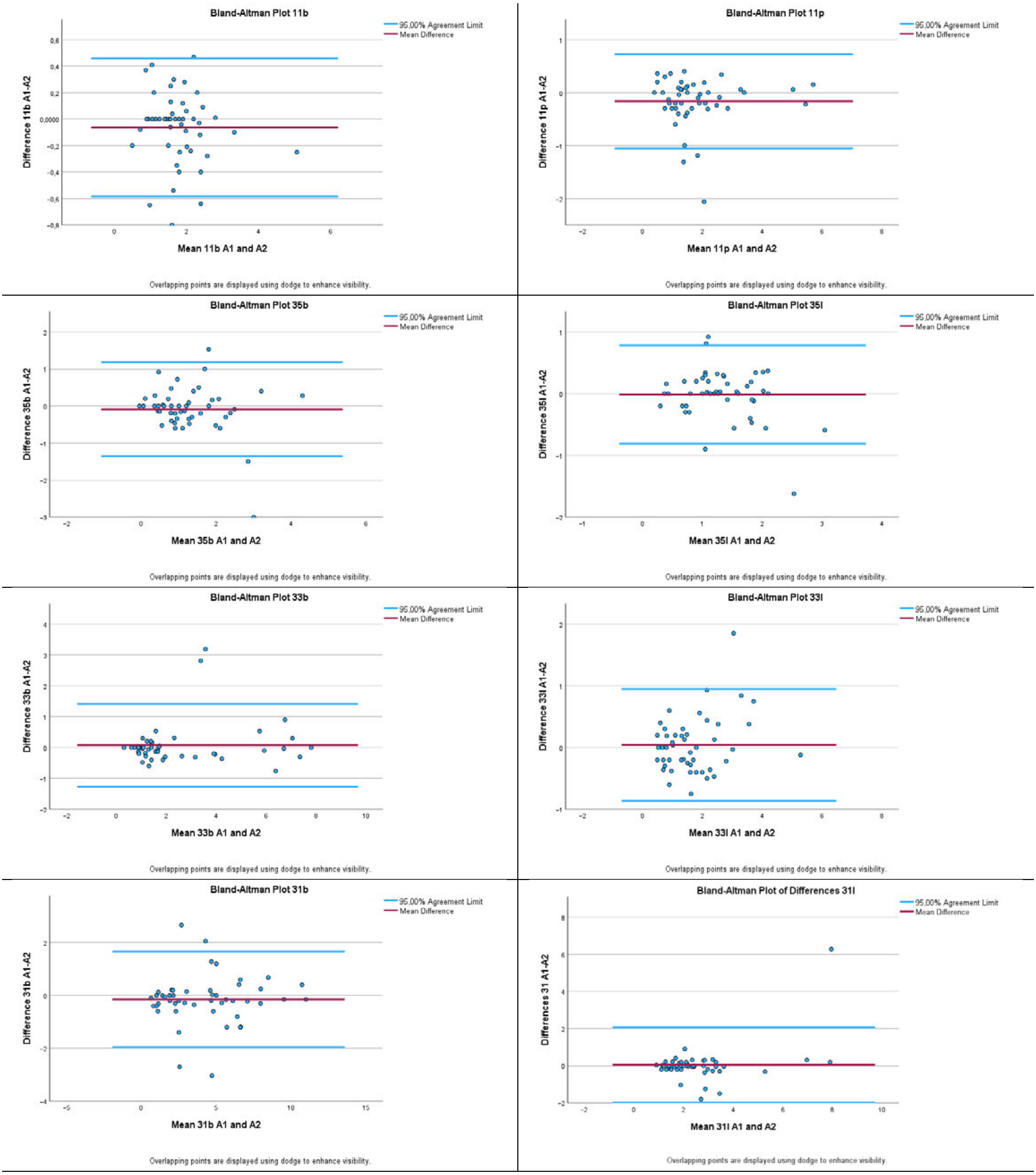

Abbreviations: A1, rater A first measurements; A2, rater A repeated measurements; b, buccal; p, palatal; l, lingual; T0, baseline; T1, post active treatment; mm, millimetres.

Note: T0 measurements were acquired from 47 CBCT volumes, and T1 measurements from 48 CBCT volumes.
