# Supplementary material for: Reliability and agreement of CBCT-based alveolar bone assessments for follow-up studies on adolescent orthodontic patients using multiplanar reconstruction and various CBCT units
Source: Prog Orthod. 2026 Jul 21;27:35. doi: 10.1186/s40510-026-00637-y (PMC13388868; doi:10.1186/s40510-026-00637-y)
Supplement: Supplementary file 2 — Supplementary Material 2. [file 40510_2026_637_MOESM2_ESM.pdf]

Additional file 2. Sensitivity analysis of outliers on Bland-Altman plots for repeated measurements (mm) of marginal bone levels by measurement site at T0 and T1

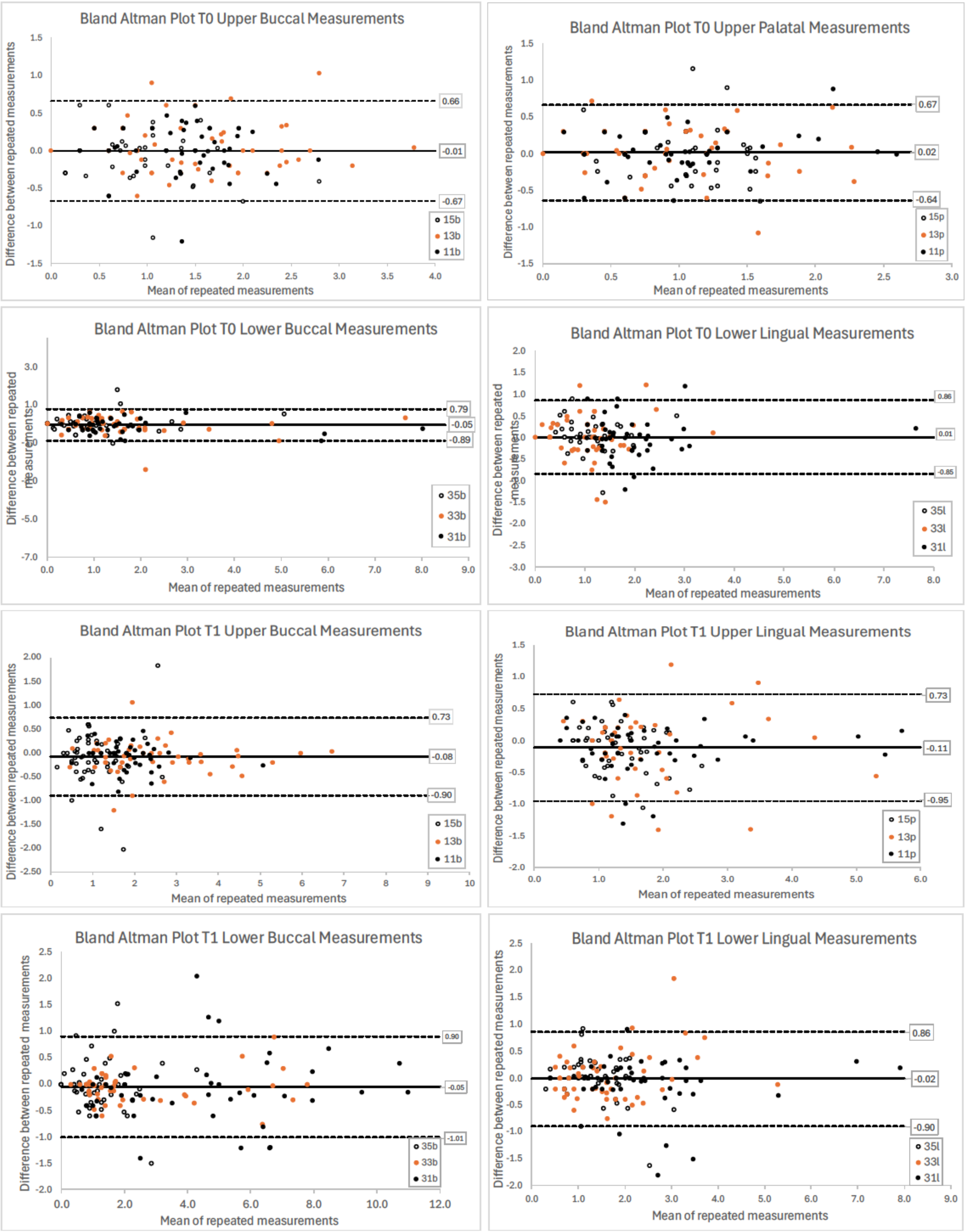

Note: Outliers exceeding three standard deviations were excluded. At T0, three outliers were excluded from each of the upper buccal and palatal plots, three from the lower buccal plot, and two from the lingual plot. At T1, three outliers were excluded from the upper buccal plot, four from the palatal plot, six from the lower buccal plot, and one from the lower lingual plot.
