# Supplementary material for: Reliability and agreement of CBCT-based alveolar bone assessments for follow-up studies on adolescent orthodontic patients using multiplanar reconstruction and various CBCT units
Source: Prog Orthod. 2026 Jul 21;27:35. doi: 10.1186/s40510-026-00637-y (PMC13388868; doi:10.1186/s40510-026-00637-y)
Supplement: Supplementary file 3 — Supplementary Material 3. [file 40510_2026_637_MOESM3_ESM.docx]

**Additional file 3**. Contingency tables underlying the agreement analyses for fenestration detection at T0 and T1 (presented in Figure 3). Numbers within the tables represent the number of assessed sites.

# Intrarater agreement results at T0

## Supplementary Table S1. Intrarater agreement for fenestration detection at upper 15b at T0

|  | Assessment B: No | Assessment B: Yes | Total |
| --- | --- | --- | --- |
| Assessment A: No | 45 | 0 | 45 |
| Assessment A: Yes | 0 | 1 | 1 |
| Total | 45 | 1 | 46 |

Observed agreement = 100 %

## Supplementary Table S1. Intrarater agreement for fenestration detection at upper 15p at T0

|  | Assessment B: No | Assessment B: Yes | Total |
| --- | --- | --- | --- |
| Assessment A: No | 46 | 0 | 46 |
| Assessment A: Yes | 0 | 0 | 0 |
| Total | 46 | 0 | 46 |

Observed agreement = 100 %

## Supplementary Table S1. Intrarater agreement for fenestration detection at upper 13b at T0

|  | Assessment B: No | Assessment B: Yes | Total |
| --- | --- | --- | --- |
| Assessment A: No | 29 | 3 | 32 |
| Assessment A: Yes | 2 | 13 | 15 |
| Total | 31 | 16 | 47 |

Observed agreement = 89 %

## Supplementary Table S1. Intrarater agreement for fenestration detection at upper 13p at T0

|  | Assessment B: No | Assessment B: Yes | Total |
| --- | --- | --- | --- |
| Assessment A: No | 46 | 0 | 46 |
| Assessment A: Yes | 1 | 0 | 1 |
| Total | 47 | 0 | 47 |

Observed agreement = 98 %

## Supplementary Table S1. Intrarater agreement for fenestration detection at upper 11b at T0

|  | Assessment B: No | Assessment B: Yes | Total |
| --- | --- | --- | --- |
| Assessment A: No | 36 | 4 | 40 |
| Assessment A: Yes | 2 | 5 | 7 |
| Total | 38 | 9 | 47 |

Observed agreement = 87 %

## Supplementary Table S1. Intrarater agreement for fenestration detection at upper 11p at T0

|  | Assessment B: No | Assessment B: Yes | Total |
| --- | --- | --- | --- |
| Assessment A: No | 47 | 0 | 47 |
| Assessment A: Yes | 0 | 0 | 0 |
| Total | 47 | 0 | 47 |

Observed agreement = 100 %

## Supplementary Table S1. Intrarater agreement for fenestration detection at lower 35b at T0

|  | Assessment B: No | Assessment B: Yes | Total |
| --- | --- | --- | --- |
| Assessment A: No | 46 | 0 | 46 |
| Assessment A: Yes | 0 | 0 | 0 |
| Total | 46 | 0 | 46 |

Observed agreement = 100 %

## Supplementary Table S1. Intrarater agreement for fenestration detection at lower 35l at T0

|  | Assessment B: No | Assessment B: Yes | Total |
| --- | --- | --- | --- |
| Assessment A: No | 46 | 0 | 46 |
| Assessment A: Yes | 0 | 0 | 0 |
| Total | 46 | 0 | 46 |

Observed agreement = 100 %

## Supplementary Table S1. Intrarater agreement for fenestration detection at lower 33b at T0

|  | Assessment B: No | Assessment B: Yes | Total |
| --- | --- | --- | --- |
| Assessment A: No | 28 | 7 | 35 |
| Assessment A: Yes | 2 | 10 | 12 |
| Total | 30 | 17 | 47 |

Observed agreement = 81 %

## Supplementary Table S1. Intrarater agreement for fenestration detection at lower 33l at T0

|  | Assessment B: No | Assessment B: Yes | Total |
| --- | --- | --- | --- |
| Assessment A: No | 47 | 0 | 47 |
| Assessment A: Yes | 0 | 0 | 0 |
| Total | 47 | 0 | 47 |

Observed agreement = 100 %

## Supplementary Table S1. Intrarater agreement for fenestration detection at lower 31b at T0

|  | Assessment B: No | Assessment B: Yes | Total |
| --- | --- | --- | --- |
| Assessment A: No | 37 | 2 | 39 |
| Assessment A: Yes | 2 | 6 | 8 |
| Total | 39 | 8 | 47 |

Observed agreement = 91 %

## Supplementary Table S1. Intrarater agreement for fenestration detection at lower 31l at T0

|  | Assessment B: No | Assessment B: Yes | Total |
| --- | --- | --- | --- |
| Assessment A: No | 46 | 0 | 46 |
| Assessment A: Yes | 0 | 1 | 1 |
| Total | 46 | 1 | 47 |

Observed agreement = 100 %

# Interrater agreement results at T0

## Supplementary Table S1. Interrater agreement for fenestration detection at upper 15b at T0

|  | Assessment B: No | Assessment B: Yes | Total |
| --- | --- | --- | --- |
| Assessment A: No | 45 | 0 | 45 |
| Assessment A: Yes | 0 | 1 | 1 |
| Total | 45 | 1 | 46 |

Observed agreement = 100 %

## Supplementary Table S1. Interrater agreement for fenestration detection at upper 15p at T0

|  | Assessment B: No | Assessment B: Yes | Total |
| --- | --- | --- | --- |
| Assessment A: No | 46 | 0 | 46 |
| Assessment A: Yes | 0 | 0 | 46 |
| Total | 46 | 0 | 46 |

Observed agreement = 100 %

## Supplementary Table S1. Interrater agreement for fenestration detection at upper 13b at T0

|  | Assessment B: No | Assessment B: Yes | Total |
| --- | --- | --- | --- |
| Assessment A: No | 24 | 8 | 32 |
| Assessment A: Yes | 4 | 11 | 15 |
| Total | 28 | 19 | 47 |

Observed agreement = 74 %

## Supplementary Table S1. Interrater agreement for fenestration detection at upper 13p at T0

|  | Assessment B: No | Assessment B: Yes | Total |
| --- | --- | --- | --- |
| Assessment A: No | 46 | 0 | 46 |
| Assessment A: Yes | 1 | 0 | 1 |
| Total | 47 | 0 | 47 |

Observed agreement = 98 %

## Supplementary Table S1. Interrater agreement for fenestration detection at upper 11b at T0

|  | Assessment B: No | Assessment B: Yes | Total |
| --- | --- | --- | --- |
| Assessment A: No | 36 | 4 | 40 |
| Assessment A: Yes | 3 | 4 | 7 |
| Total | 39 | 8 | 47 |

Observed agreement = 85 %

## Supplementary Table S1. Interrater agreement for fenestration detection at upper 11p at T0

|  | Assessment B: No | Assessment B: Yes | Total |
| --- | --- | --- | --- |
| Assessment A: No | 47 | 0 | 47 |
| Assessment A: Yes | 0 | 0 | 0 |
| Total | 47 | 0 | 47 |

Observed agreement = 100 %

## Supplementary Table S1. Interrater agreement for fenestration detection at lower 35b at T0

|  | Assessment B: No | Assessment B: Yes | Total |
| --- | --- | --- | --- |
| Assessment A: No | 45 | 1 | 46 |
| Assessment A: Yes | 0 | 0 | 0 |
| Total | 45 | 1 | 46 |

Observed agreement = 98 %

## Supplementary Table S1. Interrater agreement for fenestration detection at lower 35l at T0

|  | Assessment B: No | Assessment B: Yes | Total |
| --- | --- | --- | --- |
| Assessment A: No | 46 | 0 | 46 |
| Assessment A: Yes | 0 | 0 | 0 |
| Total | 46 | 0 | 46 |

Observed agreement = 100 %

## Supplementary Table S1. Interrater agreement for fenestration detection at lower 33b at T0

|  | Assessment B: No | Assessment B: Yes | Total |
| --- | --- | --- | --- |
| Assessment A: No | 28 | 7 | 35 |
| Assessment A: Yes | 4 | 8 | 12 |
| Total | 32 | 15 | 47 |

Observed agreement = 77 %

## Supplementary Table S1. Interrater agreement for fenestration detection at lower 33l at T0

|  | Assessment B: No | Assessment B: Yes | Total |
| --- | --- | --- | --- |
| Assessment A: No | 47 | 0 | 47 |
| Assessment A: Yes | 0 | 0 | 0 |
| Total | 47 | 0 | 47 |

Observed agreement = 100 %

## Supplementary Table S1. Interrater agreement for fenestration detection at lower 31b at T0

|  | Assessment B: No | Assessment B: Yes | Total |
| --- | --- | --- | --- |
| Assessment A: No | 33 | 6 | 39 |
| Assessment A: Yes | 4 | 4 | 8 |
| Total | 37 | 10 | 47 |

Observed agreement = 79 %

## Supplementary Table S1. Interrater agreement for fenestration detection at lower 31l at T0

|  | Assessment B: No | Assessment B: Yes | Total |
| --- | --- | --- | --- |
| Assessment A: No | 46 | 0 | 46 |
| Assessment A: Yes | 1 | 0 | 1 |
| Total | 47 | 0 | 47 |

Observed agreement = 98 %

# Intrarater agreement results at T1

## Supplementary Table S1. Intrarater agreement for fenestration detection at upper 15b at T1

|  | Assessment B: No | Assessment B: Yes | Total |
| --- | --- | --- | --- |
| Assessment A: No | 41 | 1 | 42 |
| Assessment A: Yes | 1 | 5 | 6 |
| Total | 42 | 6 | 48 |

Observed agreement = 96 %

## Supplementary Table S1. Intrarater agreement for fenestration detection at upper 15p at T1

|  | Assessment B: No | Assessment B: Yes | Total |
| --- | --- | --- | --- |
| Assessment A: No | 48 | 0 | 48 |
| Assessment A: Yes | 0 | 0 | 0 |
| Total | 48 | 0 | 48 |

Observed agreement = 100 %

## Supplementary Table S1. Intrarater agreement for fenestration detection at upper 13b at T1

|  | Assessment B: No | Assessment B: Yes | Total |
| --- | --- | --- | --- |
| Assessment A: No | 31 | 3 | 34 |
| Assessment A: Yes | 3 | 11 | 14 |
| Total | 34 | 14 | 48 |

Observed agreement = 88 %

## Supplementary Table S1. Intrarater agreement for fenestration detection at upper 13p at T1

|  | Assessment B: No | Assessment B: Yes | Total |
| --- | --- | --- | --- |
| Assessment A: No | 47 | 0 | 47 |
| Assessment A: Yes | 1 | 0 | 1 |
| Total | 48 | 0 | 48 |

Observed agreement = 98%

## Supplementary Table S1. Intrarater agreement for fenestration detection at upper 11b at T1

|  | Assessment B: No | Assessment B: Yes | Total |
| --- | --- | --- | --- |
| Assessment A: No | 45 | 0 | 45 |
| Assessment A: Yes | 1 | 2 | 3 |
| Total | 46 | 2 | 48 |

Observed agreement = 98 %

## Supplementary Table S1. Intrarater agreement for fenestration detection at upper 11p at T1

|  | Assessment B: No | Assessment B: Yes | Total |
| --- | --- | --- | --- |
| Assessment A: No | 46 | 1 | 47 |
| Assessment A: Yes | 1 | 0 | 1 |
| Total | 47 | 1 | 48 |

Observed agreement = 96 %

## Supplementary Table S1. Intrarater agreement for fenestration detection at lower 35b at T1

|  | Assessment B: No | Assessment B: Yes | Total |
| --- | --- | --- | --- |
| Assessment A: No | 45 | 3 | 48 |
| Assessment A: Yes | 0 | 0 | 0 |
| Total | 45 | 3 | 48 |

Observed agreement = 94 %

## Supplementary Table S1. Intrarater agreement for fenestration detection at lower 35l at T1

|  | Assessment B: No | Assessment B: Yes | Total |
| --- | --- | --- | --- |
| Assessment A: No | 48 | 0 | 48 |
| Assessment A: Yes | 0 | 0 | 0 |
| Total | 48 | 0 | 48 |

Observed agreement = 100 %

## Supplementary Table S1. Intrarater agreement for fenestration detection at lower 31b at T1

|  | Assessment B: No | Assessment B: Yes | Total |
| --- | --- | --- | --- |
| Assessment A: No | 34 | 2 | 36 |
| Assessment A: Yes | 1 | 11 | 12 |
| Total | 35 | 13 | 48 |

Observed agreement = 94 %

## Supplementary Table S1. Intrarater agreement for fenestration detection at lower 31l at T1

|  | Assessment B: No | Assessment B: Yes | Total |
| --- | --- | --- | --- |
| Assessment A: No | 47 | 0 | 47 |
| Assessment A: Yes | 0 | 1 | 1 |
| Total | 47 | 1 | 48 |

Observed agreement = 100%

## Supplementary Table S1. Intrarater agreement for fenestration detection at lower 31b at T1

|  | Assessment B: No | Assessment B: Yes | Total |
| --- | --- | --- | --- |
| Assessment A: No | 46 | 1 | 47 |
| Assessment A: Yes | 0 | 1 | 1 |
| Total | 46 | 2 | 48 |

Observed agreement = 98 %

## Supplementary Table S1. Intrarater agreement for fenestration detection at lower 31l at T1

|  | Assessment B: No | Assessment B: Yes | Total |
| --- | --- | --- | --- |
| Assessment A: No | 46 | 0 | 46 |
| Assessment A: Yes | 1 | 1 | 2 |
| Total | 47 | 1 | 48 |

Observed agreement = 98 %

# Interrater agreement results at T1

## Supplementary Table S1. Interrater agreement for fenestration detection at upper 15b at T1

|  | Assessment B: No | Assessment B: Yes | Total |
| --- | --- | --- | --- |
| Assessment A: No | 41 | 1 | 42 |
| Assessment A: Yes | 2 | 4 | 6 |
| Total | 43 | 5 | 48 |

Observed agreement = 94 %

## Supplementary Table S1. Interrater agreement for fenestration detection at upper 15p at T1

|  | Assessment B: No | Assessment B: Yes | Total |
| --- | --- | --- | --- |
| Assessment A: No | 48 | 0 | 48 |
| Assessment A: Yes | 0 | 0 | 0 |
| Total | 48 | 0 | 48 |

Observed agreement = 100 %

## Supplementary Table S1. Interrater agreement for fenestration detection at upper 13b at T1

|  | Assessment B: No | Assessment B: Yes | Total |
| --- | --- | --- | --- |
| Assessment A: No | 28 | 6 | 34 |
| Assessment A: Yes | 5 | 9 | 14 |
| Total | 33 | 15 | 48 |

Observed agreement = 77 %

## Supplementary Table S1. Interrater agreement for fenestration detection at upper 13p at T1

|  | Assessment B: No | Assessment B: Yes | Total |
| --- | --- | --- | --- |
| Assessment A: No | 47 | 0 | 47 |
| Assessment A: Yes | 1 | 0 | 1 |
| Total | 48 | 0 | 48 |

Observed agreement = 98%

## Supplementary Table S1. Interrater agreement for fenestration detection at upper 11b at T1

|  | Assessment B: No | Assessment B: Yes | Total |
| --- | --- | --- | --- |
| Assessment A: No | 40 | 5 | 45 |
| Assessment A: Yes | 1 | 2 | 3 |
| Total | 41 | 7 | 48 |

Observed agreement = 86 %

## Supplementary Table S1. Interrater agreement for fenestration detection at upper 11p at T1

|  | Assessment B: No | Assessment B: Yes | Total |
| --- | --- | --- | --- |
| Assessment A: No | 45 | 2 | 47 |
| Assessment A: Yes | 1 | 0 | 1 |
| Total | 46 | 2 | 48 |

Observed agreement = 94 %

## Supplementary Table S1. Interrater agreement for fenestration detection at lower 35b at T1

|  | Assessment B: No | Assessment B: Yes | Total |
| --- | --- | --- | --- |
| Assessment A: No | 44 | 4 | 48 |
| Assessment A: Yes | 0 | 0 | 0 |
| Total | 44 | 4 | 48 |

Observed agreement = 92%

## Supplementary Table S1. Interrater agreement for fenestration detection at lower 35l at T1

|  | Assessment B: No | Assessment B: Yes | Total |
| --- | --- | --- | --- |
| Assessment A: No | 47 | 1 | 48 |
| Assessment A: Yes | 0 | 0 | 0 |
| Total | 47 | 1 | 48 |

Observed agreement = 98 %

## Supplementary Table S1. Interrater agreement for fenestration detection at lower 33b at T1

|  | Assessment B: No | Assessment B: Yes | Total |
| --- | --- | --- | --- |
| Assessment A: No | 28 | 8 | 36 |
| Assessment A: Yes | 3 | 9 | 12 |
| Total | 31 | 17 | 48 |

Observed agreement = 77 %

## Supplementary Table S1. Interrater agreement for fenestration detection at lower 33l at T1

|  | Assessment B: No | Assessment B: Yes | Total |
| --- | --- | --- | --- |
| Assessment A: No | 47 | 0 | 47 |
| Assessment A: Yes | 1 | 0 | 1 |
| Total | 48 | 0 | 48 |

Observed agreement = 98%

## Supplementary Table S1. Interrater agreement for fenestration detection at lower 31b at T1

|  | Assessment B: No | Assessment B: Yes | Total |
| --- | --- | --- | --- |
| Assessment A: No | 43 | 4 | 47 |
| Assessment A: Yes | 1 | 0 | 1 |
| Total | 44 | 4 | 48 |

Observed agreement = 90%

## Supplementary Table S1. Interrater agreement for fenestration detection at lower 31l at T1

|  | Assessment B: No | Assessment B: Yes | Total |
| --- | --- | --- | --- |
| Assessment A: No | 46 | 0 | 46 |
| Assessment A: Yes | 2 | 0 | 2 |
| Total | 48 | 0 | 48 |

Observed agreement = 96%
